# Genome-wide analysis of alternative splicing in *Volvox carteri*

**DOI:** 10.1186/1471-2164-15-1117

**Published:** 2014-12-16

**Authors:** Arash Kianianmomeni, Cheng Soon Ong, Gunnar Rätsch, Armin Hallmann

**Affiliations:** Department of Cellular and Developmental Biology of Plants, University of Bielefeld, Universitätsstr. 25, D-33615 Bielefeld, Germany; Machine Learning Group, NICTA Canberra Research Laboratory, Canberra, Australia; Biomedical Data Science Group, Memorial Sloan-Kettering Cancer Center, 1275 York Avenue, New York City, NY 10065 USA

**Keywords:** Bioinformatics, Differential splicing, EST analysis, Green algae, Lower eukaryotes, Quantitative real-time RT-PCR, Transcriptome

## Abstract

**Background:**

Alternative splicing is an essential mechanism for increasing transcriptome and proteome diversity in eukaryotes. Particularly in multicellular eukaryotes, this mechanism is involved in the regulation of developmental and physiological processes like growth, differentiation and signal transduction.

**Results:**

Here we report the genome-wide analysis of alternative splicing in the multicellular green alga *Volvox carteri*. The bioinformatic analysis of 132,038 expressed sequence tags (ESTs) identified 580 alternative splicing events in a total of 426 genes. The predominant type of alternative splicing in *Volvox* is intron retention (46.5%) followed by alternative 5′ (17.9%) and 3′ (21.9%) splice sites and exon skipping (9.5%). Our analysis shows that in *Volvox* at least ~2.9% of the intron-containing genes are subject to alternative splicing. Considering the total number of sequenced ESTs, the *Volvox* genome seems to provide more favorable conditions (e.g., regarding length and GC content of introns) for the occurrence of alternative splicing than the genome of its close unicellular relative *Chlamydomonas*. Moreover, many randomly chosen alternatively spliced genes of *Volvox* do not show alternative splicing in *Chlamydomonas*. Since the *Volvox* genome contains about the same number of protein-coding genes as the *Chlamydomonas* genome (~14,500 protein-coding genes), we assumed that alternative splicing may play a key role in generation of genomic diversity, which is required to evolve from a simple one-cell ancestor to a multicellular organism with differentiated cell types (Mol Biol Evol 31:1402-1413, 2014). To confirm the alternative splicing events identified by bioinformatic analysis, several genes with different types of alternatively splicing have been selected followed by experimental verification of the predicted splice variants by RT-PCR.

**Conclusions:**

The results show that our approach for prediction of alternative splicing events in *Volvox* was accurate and reliable. Moreover, quantitative real-time RT-PCR appears to be useful in *Volvox* for analyses of relationships between the appearance of specific alternative splicing variants and different kinds of physiological, metabolic and developmental processes as well as responses to environmental changes.

**Electronic supplementary material:**

The online version of this article (doi:10.1186/1471-2164-15-1117) contains supplementary material, which is available to authorized users.

## Background

Alternative splicing of precursor messenger RNA (pre-mRNA) is an important post-transcriptional regulatory mechanism that enhances the transcriptome plasticity and proteome diversity. Alternative splicing produces multiple transcripts from a single gene by varying the selection of the include/exclude regions. The different splicing products of a single gene produce different protein isoforms with different functions and effects [1–10]. Alternative splicing can also introduce premature stop codons, which cause down-regulation of expression of the corresponding gene by nonsense-mediated decay (NMD) of mRNA [11].

The five basic types of alternative splicing are as follows: 1) exon skipping, if an exon is either included in or excluded from the pre-mRNA; 2) intron retention, when an intron is either retained or excised from the pre-mRNA; 3) alternative 5′ splice sites and 4) alternative 3′ splices site allow the extension or shortening of a particular exon, depending on the use of a proximal or distal 5′ and 3′ splice site, respectively; 5) mutually exclusive exons occur when two or more adjacent cassette exons are spliced such that only one of them is included at a time in the mRNA [2, 5, 12].

Alternative splicing events are not rare, but quite common in eukaryotes. In human, ~95% of all intron-containing genes are alternatively spliced, ~60% in *Drosophila melanogaster*, ~25% in *Caenorhabditis elegans* and ~61% in *Arabidopsis thaliana* (hereafter *Arabidopsis*) [13–21]. The real percentages might even be higher than reported, because they correlate with the number of sequenced ESTs. An increased number of sequenced ESTs frequently reveal additional alternative splicing events because more and more rare splicing variants from genes with low expression become sequenced. For *Arabidopsis* the reported percentage of alternatively spliced genes increased dramatically within a decade: it was 1.2% in 2003 [22], 11.6% in 2004 [23], more than 30% in 2006 [24], 42% in 2010 [18] and 61% in 2012 [19].

In *Arabidopsis*, the most frequent alternative splicing variant is intron retention (~40%) [18, 19]. Most alternative splicing events in *Arabidopsis*, i.e. 78.4%, occur in the coding region and about 50% of which produce a premature termination codon that is a potential target for NMD [5, 25]. In addition, 15.2% of all alternative splicing events occur in the 5′-untranslated region (UTR) and 6.4% in the 3′ UTR [5]. In humans, the allocation is quite different from *Arabidopsis*: the most common alternative splicing variant is exon skipping (42-58%), whereas intron retention forms only a small fraction (5-9%) of all alternative splicing events [5, 26].

Two main factors that affect the occurrence of alternative splicing are intron lengths and the nucleotide composition of the introns [27]. Intronic nucleotide composition has been shown to affect splicing efficiency of intron retention [10, 28–30]. Compared to the average length of human introns, which is 3365 bp, *Arabidopsis* introns are much shorter and show an average length of only 170 bp [31, 32]. In human introns the AT content is only 51.9% [32], while plant introns show a high AT content: in *Arabidopsis* it is 67% and in rice it is 73% [5, 33, 34]. Moreover, the nucleotide composition of plant introns is also different between dicots and monocots. In rice, for example, the introns are longer and have a higher GC content than in *Arabidopsis*, which might be an indication for a different impact of alternative splicing in these organisms [27, 35–37].

Alternative splicing produces protein isoforms that differ from each other with regard to localization, enzymatic activity, signaling effects and protein stability [2–5]. In plants, alternative splicing was shown to be involved in signal transduction and timing of flowering [5]. Alternative splicing can also act as a gene regulatory mechanism during developmental processes or in response to environmental conditions [2–6, 8, 9]. Various biotic and abiotic stress factors are known to influence alternative splicing [4, 6, 38–41]. Relevant abiotic stress factors are heavy metals, cold and heat. For example, the splicing of polyubiquitin and *hsp70* mRNAs in maize is affected by a heat shock [42, 43]. Biotic stress factors that influence alternative splicing are viral and bacterial pathogens [5, 44, 45]. Plants even seem to regulate their transcriptome post-transcriptionally in response to quickly changing environmental conditions and pathogen attacks by using alternative splicing mechanisms [39, 46, 47].

Like in higher plants and animals, alternative splicing also is a common mechanism for increasing transcriptome diversity in much simpler organisms like algae. Previous studies in volvocine green algae, which include unicellular forms like *Chlamydomonas reinhardtii* (hereafter *Chlamydomonas*) to colonial and multicellular forms with increasing complexity like *Volvox carteri* (hereafter *Volvox*), revealed a number of genes that undergo alternative splicing. Examples include *algal-CAM* [48] and *RBR1*/*mat3* [49, 50] in *Volvox* and *Cop1* [51] and *CGE1* [52] in *Chlamydomonas*. A recent study about alternative splicing in *Chlamydomonas* indicates that about 3% of all genes in *Chlamydomonas* undergo alternative splicing [53], which is much lower than recent reports from higher plants (e.g., 61% in *Arabidopsis*; based on the analysis of 116 million paired-end RNA-seq reads of a normalized cDNA library) [18, 19]. The analysis of a large EST dataset of *Chlamydomonas* resulted in 498 EST clusters that show 611 alternative splicing events [53]. The results indicated that 11.6% of the alternative splicing events in *Chlamydomonas* (based on the analysis of 252,484 ESTs) are alternative 5′ splice sites, 25.8% are alternative 3′ splice sites, 0.7% show both alternative 5′ and 3′ splice sites and 11.9% show exon skipping. Like in *Arabidopsis*, the most frequent alternative splicing event in *Chlamydomonas* is intron retention, which accounts for 50% of all events [53].

Based on molecular-phylogenetic studies, *Volvox* and *Chlamydomonas* probably diverged ~ 200 million years ago from a common unicellular ancestor [54]. On the time-scales of evolution, the transition from unicellular to multicellular life in *Volvox* is thus a quite recent occurrence when compared to other shifts to multicellularity. Other transitions to multicellularity, such as the ones that gave rise to plants and animals, occurred deep in the past, approaching a billion years ago [55, 56]. The evolution of multicellular live in volvocine algae required several developmental traits including asymmetric cell division and embryonic morphogenesis. Most probably, the first multicellular volvocine algae were just small colonial organisms (like *Gonium*) without differentiated cells. Later size, cell number and overall complexity increased and a tendency to cell differentiation evolved (like in *Eudorina* and *Pleodorina*). Finally, even a complete division of labor between somatic cells and germ cells developed (like in *Volvox*) [57]. Comparative analyses of the *Volvox* and *Chlamydomonas* genomes revealed that the overall sequence divergence between these organisms is comparable to that between human and chicken (which diverged ~310 million years ago) and *Arabidopsis* and poplar (which diverged ~110 million years ago). Moreover, despite conserved synteny between the genomes, *Volvox* and *Chlamyomdonas* show higher rates of genomic rearrangement than vertebrates and eudicots do [58]. The nuclear genome of *Chlamydomonas* is 118 Mbp in size and that of its multicellular relative *Volvox* is composed of 138 Mbp. The larger genome of *Volvox* (~17% larger) is attributed to its higher content of transposons and repetitive DNA [58, 59] because both species have almost identical protein-coding potentials, i.e., 14,516 and 14,520 protein-coding genes in *Chlamydomonas* and *Volvox*, respectively. Only a few gene families, i.e., the pherophorin genes, the VMP genes (*Volvox* matrix metalloproteases) and the cyclin-D related genes have more members in *Volvox* than in *Chlamydomonas* [58]. This suggests that the transition from a unicellular, *Chlamydomonas*-like ancestor to multicellular *Volvox* did not take major changes in gene content [58, 60] but mainly alterations in the mechanisms of genetic regulation. Thus, development of organismal complexity might be mainly caused by evolutionary innovations of pre-existing proteins (e.g., transcription factors) and their binding sites, inventions of noncoding RNAs, innovations in the mechanism of alternative splicing and increase of alternative splicing events [58, 61–64]. Alternative splicing can produce different protein isoforms from a single gene, which has produced only a single protein in an ancestor; in this way, diversity increases. Together with differences in selection pressures within a population, appearance or changes in alternative splicing can lead to speciation.

Interestingly, two key factors that affect the splicing mechanism are different between the genomes of *Chlamydomonas* and *Volvox*: the intron length and the nucleotide composition. With an average length of 491 bp, the introns of *Volvox* are clearly larger than *Chlamydomonas* introns, which span only 371 bp on average [58] (Additional file 1: Table S1). Bioinformatic analyses showed that exons flanked by longer introns are more frequently subject to alternative splicing events than exons flanked by short introns [65, 66]. Furthermore, the genome of *Volvox* shows a lower GC content (56%) than the genome of *Chlamydomonas* (64%) [58, 59], which might cause differences between the two species in alternative splicing [27, 35]. Based on these two key differences between both genomes, a detailed investigation of alternative splicing may reveal new insights into the gene regulation mechanisms that have been required for the evolutionary transition from unicellular *Chlamydomonas* to multicellular *Volvox*, while the number of genes remained about the same during this transition.

Here we report the analysis of alternative splicing in the multicellular green alga *Volvox*. After bioinformatic analysis of 132,038 ESTs, we identified 580 alternative splicing events corresponding to 426 genes. We show the distribution of the different types of alternative splicing events in *Volvox* and compare it with other species. To confirm our bioinformatic results, several alternatively spliced genes have been selected as representatives for experimental verification. After confirmation of alternative splicing variants by reverse transcription polymerase chain reaction (RT-PCR), the relative expression level of each splice variant was determined using quantitative real-time RT-PCR.

Our results indicate that alternative splicing is a widespread process for generating protein isoform diversity in *Volvox*, which suggests an important role of alternative splicing for expansion of organismal complexity during evolution of multicellularity and cell differentiation in volvocine algae.

## Results

### Genomic mapping of ESTs

The genome-wide analysis of alternative splicing in *Volvox* and its comparison with both a closely related unicellular alga (*Chlamydomonas*) and a more distantly related higher plant (*Arabidopsis*) required both extensive genomic and EST sequence data. These data were obtained from the corresponding databases of *Volvox* [58], *Chlamydomonas* [59] and *Arabidopsis* [31] (see Methods). The data sets of the three species were treated in the same way to provide the necessary comparability.

All available ESTs of the three species were aligned to the corresponding genomic contigs and genome sequences using BLAT, a BLAST-like alignment tool [67]. Only the best alignment was used to avoid double counting of paralogs. EST sequences with less than 95% identity to any sequence in the corresponding genome were removed from further analysis. The resulting alignments were then clustered by their genomic location. In this process a cluster arises from the set of all ESTs, which overlap at a given genomic location.

Subsequently, the splice site consensus sequences were identified for all splicing events. The vast majority of introns in protein-coding genes of *Volvox* and of any other previously investigated eukaryote are canonical, which means that they have a GT dinucleotide at their 5′ end and an AG dinucleotide their 3′ end [68]. Only about 1-2% of the introns are non-canonical. To compensate for artefacts that may occur in further analysis, we omitted alternative splicing events that involved introns with non-canonical splice site dinucleotides (i.e., not GT/AG).

Then, alternative splicing graphs of potential splice variants were constructed for each cluster in the three genomes. An intron was constructed in a given splice graph when there was EST evidence of a transcript with canonical splice sites.

**Figure 1:**
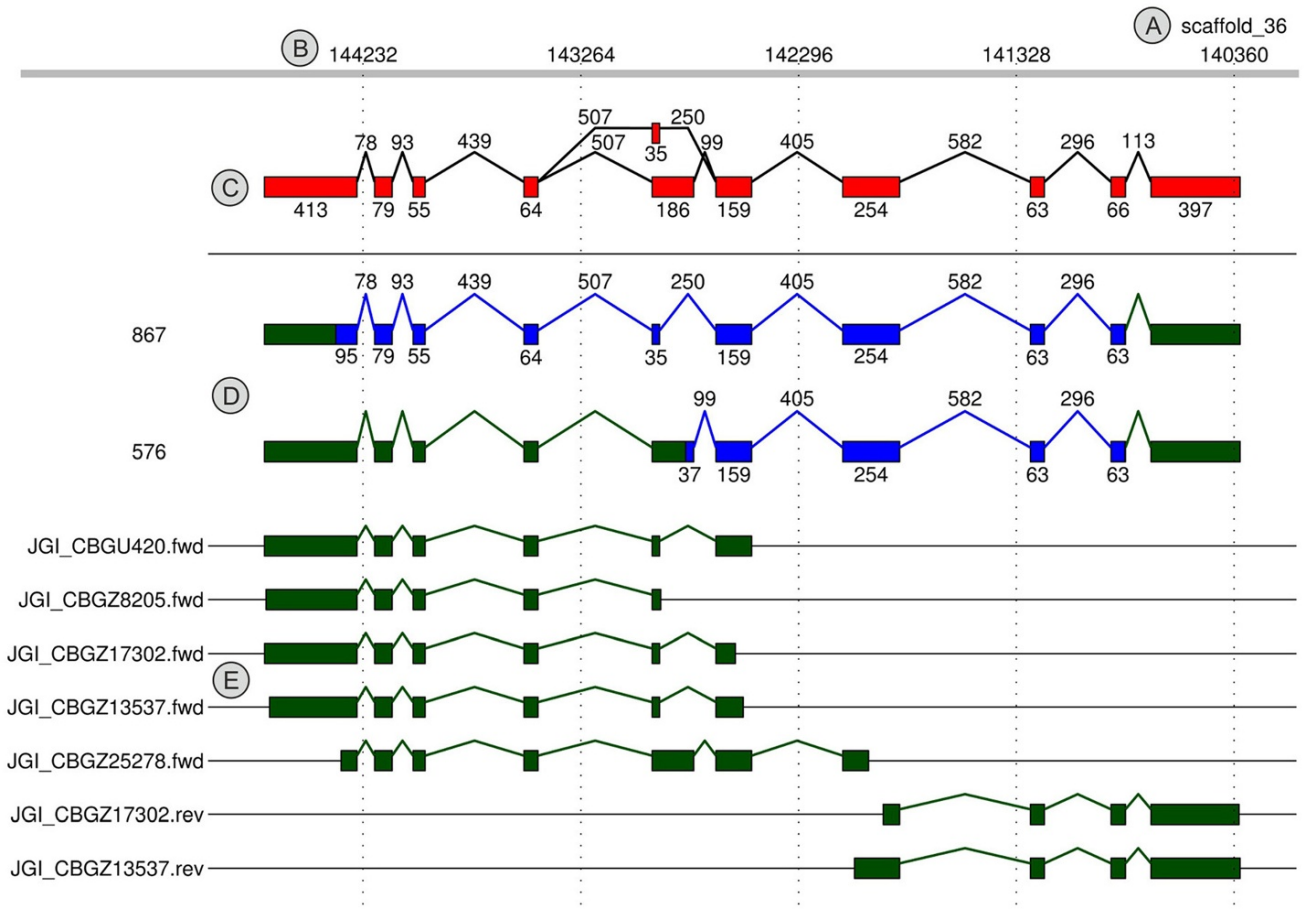
**Representative sample of one alternative splicing locus out of 6925 loci in** ***Volvox*** **.** The generated graph shows locus 3667, a gene for a serine threonine phosphatase. **(A)** Number of the corresponding genomic scaffold. **(B)** Nucleotide position within scaffold. **(C)** Cumulated graph with all splicing events at the given locus; the lengths of introns and exons are indicated. **(D)** Separate depiction of all identified splicing variants (here two variants). The longest open reading frame is shown in blue color; the length of the open reading frame is shown on the left side. **(E)** Depiction of all ESTs that were mapped to this genomic locus. The names of the ESTs (left side) are just as deposited in the GenBank EST database (http://www.ncbi.nlm.nih.gov/dbEST/).

A representative sample of one alternative splicing locus out of 6,925 loci in *Volvox* is shown in Figure 1. At the 6,925 loci we identified 31,885 exons. This gives an average of 4.6 exons per locus.

### Alternative splicing analysis and abundance of splicing events

After genomic mapping of all available ESTs, we identified the alternatively spliced isoforms for each locus in *Volvox*, *Chlamydomonas* and *Arabidopsis*. Isoforms with alternative transcription starts or ends were not considered further in this paper, because we focus here on alternative splicing events. The alternatively spliced isoforms were divided into four major groups of events: exon skipping, intron retention, alternative 5′ and alternative 3′ splicing. In addition, there were instances of more complex splice forms, which were not covered by the above four simple alternative splicing events. To obtain splicing events with a high quality, we complement our predictions by a quality value to penalize the spurious events with poor EST support.

The bioinformatic analysis identified 580 alternative splicing events in *Volvox* in a total of 426 genes. Thus, about 2.9% of all ~14,500 protein-coding *Volvox* genes are subject to alternative splicing. This percentage is more similar to the one observed in higher plants than to that in the closely related unicellular relative *Chlamydomonas*; considering the number of analyzed ESTs.

**Figure 2:**
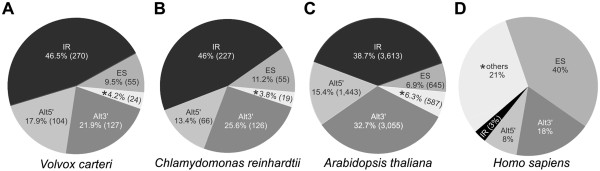
**Distribution of the different types of alternative splicing events.** The percentage and total number (in parenthesis) of splicing events is shown for each type of alternative splicing, i.e., intron retention (IR), exon skipping (ES), alternative 5′ splice site (Alt5′) and alternative 3′ splice site (Alt3′). Asterisks (*) indicate the percentage of other, more complex alternative splicing types. The distribution is given for the species *Volvox* **(A)**, *Chlamydomonas* **(B)**, *Arabidopsis* **(C)** and human **(D)**. The total number of alternative splicing events is 580 in *Volvox*, 493 in *Chlamydomonas* and 9,343 in *Arabidopsis*.

The analysis of the different types of alternative splicing revealed that 9.5% of all alternative splicing events show exon skipping in *Volvox*, 46.5% show intron retention, 17.9% alternative 5′ splice sites and 21.9% alternative 3′ splice sites (Figure 2A). Thus, the predominant type of alternative splicing in *Volvox* is intron retention followed by alternative 3′ splice sites, alternative 5′ splice sites and exon skipping.

In the closely related alga *Chlamydomonas*, the intron retention also is the most common type of alternative splicing (46%), followed by alternative 3′ splice sites (25.6%), alternative 5′ splice sites (13.4%) and exon skipping (11.2%) (Figure 2B). In *Arabidopsis*, the prevalence of different alternative splicing types shows the same distribution as in *Volvox* and *Chlamydomonas*, i.e. the percentage decreases in the following order: intron retention > alternative 3′ splice sites > alternative 5′ splice sites > exon skipping (Figure 2C). In contrast to non-plant model organisms like human (Figure 2D), exon skipping is the rarest simple form of alternative splicing in all of the three investigated species (Figures 2A-C).

### Localization of alternative splicing events

**Figure 3:**
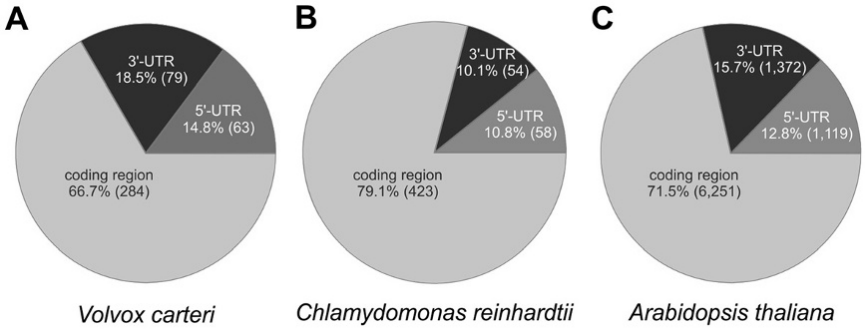
**Localization of alternative splicing events in the mRNAs.** In the species *Volvox* **(A)**, *Chlamydomonas* **(B)** and *Arabidopsis* **(C)** each mRNA was divided into coding region, 5′ UTR or 3′ UTR. The percentage and total number (in parenthesis) of splicing events is given for each of these mRNA regions. The splicing events totaled together 426 events in *Volvox*, 535 in *Chlamydomonas* and 8,742 in *Arabidopsis*.

In *Volvox*, the majority of all alternative splicing events (66.7%) affect the coding regions. Another 33.3% occur within non-coding regions (14.8% in 5′ UTRs and 18.5% in 3′ UTRs) (Figure 3A). The results from *Volvox* were again compared with the genome and EST data of *Chlamydomonas* and *Arabidopsis* [31, 59], which were treated in the same way as the data from *Volvox* (see Methods). In *Chlamydomonas*, 10.8% of the alternative splicing events were detected in 5′ UTRs and 10.1% in 3′ UTRs (Figure 3B). In *Arabidopsis*, 12.8% of the alternative splicing events were localized in 5′ UTRs and 15.7% in 3′ UTRs (Figure 3C). In both organisms, the majority of alternative splicing events occur within the coding region, just as observed in *Volvox*. More precisely, it was 79.1% in *Chlamydomonas* and 71.5% in *Arabidopsis* (Figures 3B and 3C).

### Experimental verification of alternative splicing events

To validate the quality of both the used ESTs and our bioinformatic analysis, ten sample genes with identified alternative splicing events were selected for experimental verification by RT-PCR and quantitative real time RT-PCR (Additional file 1: Table S2). The decisive factor in the choice of a sample gene to be tested was the potential modification of protein domains or protein sequence motifs by alternative splicing. Strong EST support, however, was not relevant for our selection. As a consequence of this approach, for some of the selected sample genes, like the genes for the mitochondrial translation elongation factor Tu (*efg8*) and the selenocysteine-specific elongation factor (*selEFf*) [50], only one supporting EST for a given alternative splicing variant was available. By contrast, for other genes, like the gene for the oxygen evolving enhancer protein 1 (*ooe1*), more than one hundred ESTs existed (data not shown).

**Figure 4:**
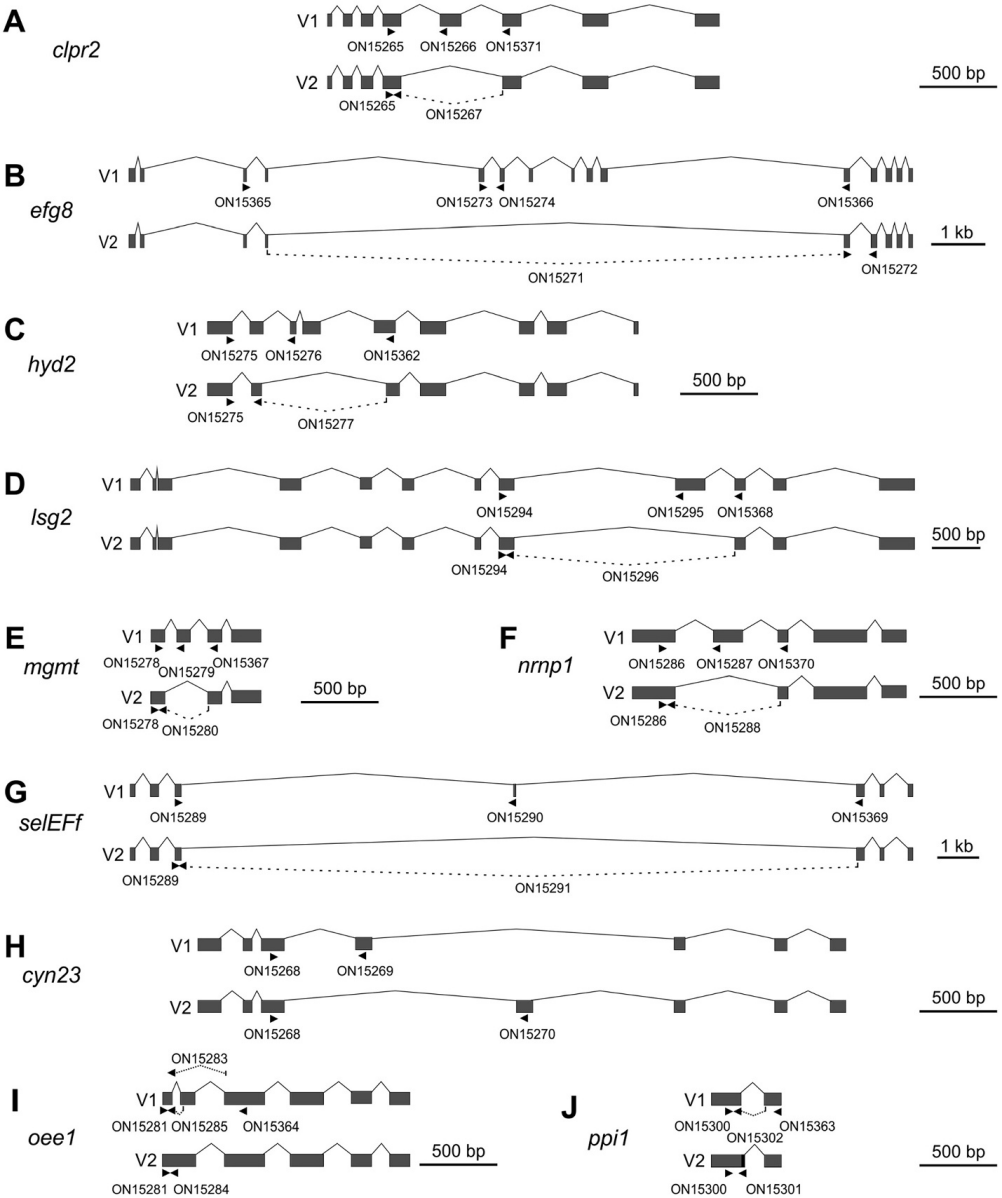
**Gene structures and alternative splicing of ten sample genes.** Two splice variants (V1 and V2) are shown for each gene. Grey boxes are exons and the carets represent introns. Arrow heads indicate the position of primers used for amplification of the respective alternative splicing variant (V1 or V2); primers used for amplification of both splice variants in one reaction tube are shown in the depiction of V1 (Additional file 1: Table S3). Dashed lines indicate that a primer spans an exon-exon junction; the exact position of the exon-exon boundary within such a primer is given in Additional file 1: Table S3. Genes that undergo exon skipping are *clpr2* **(A)**, *efg8* **(B)**, *hyd2* **(C)**, *lsg2* **(D)**, *mgmt* **(E)**, *nrnp1* **(F)** and *selEFf* **(G)**. *cyn23* **(H)** has mutually exclusive exons, *oee1* **(I)** shows intron retention and *ppi1* **(J)** uses alternative 5′ splice sites. In the second splice variant (V2) of *ppi1*, 21 bp at the 5′ side of the intron are retained (black area).

The statistical evaluation of the data obtained from our bioinformatic analysis of alternative splicing indicates that splice variants of genes that show exon skipping lead more frequently to changes in protein properties (like protein localization and activity) than genes showing any other type of alternative splicing (data not shown). For that reason, the following seven sample genes with exon skipping and (putative) differences in the properties of the protein variants were selected: *clpr2* (chloroplast Clp protease), *efg8* (mitochondrial translation elongation factor Tu), *hyd2* (iron hydrogenase), *lsg2* (matrix metalloproteinase), *mgmt* (6-O-methylguanine DNA methyltransferase), *nrnp1* (nuclear ribonucleoprotein) and *selEFf* (selenocysteine-specific elongation factor) (Figure 4 and Additional file 1: Table S2). Of particular interest was the experimental verification of the splicing products of *efg8* and *selEFf* because of their uncommon gene structures with extremely long introns, which were 10772 bp (*efg8*) and 16365 bp (*selEFf*) in length (Figure 4).

In addition to exon skipping, we chose three further sample genes with other types of alternative splicing, which also lead to changes in protein properties. The selected genes were *cyn23* (cyclophilin-type peptidyl-prolyl cis-trans isomerase), which shows alternative splicing via mutually exclusive exons, *oee1* (oxygen evolving enhancer protein 1), which is an example for intron retention, and *ppi1* (protein phosphatase inhibitor), which produces protein isoforms through alternative 5′ splice sites (Figure 4 and Additional file 1: Table S2).

To allow for a distinction of the different splice variants of a given gene, we named the variants “first” and “second” splice variant. More precisely, the variant that shows an exon-intron structure identical or similar to a gene structure predicted as part of the *Volvox* genome project [58] or in a database entry (Additional file 1: Table S2) was called “first splice variant” and another splice variant was called “second splice variant”.

#### Verification of exon skipping

**Figure 5:**
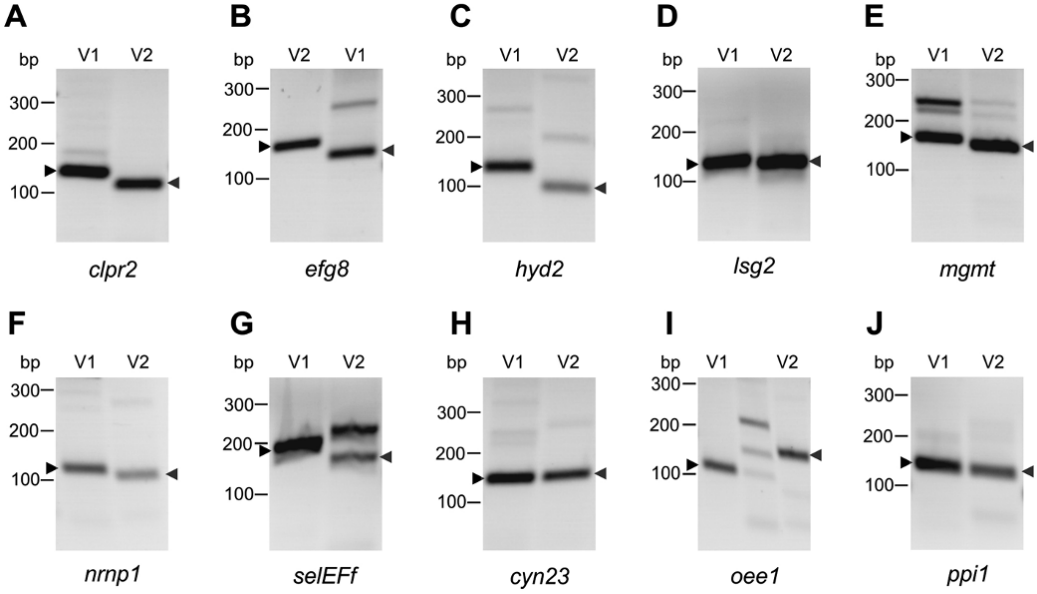
**RT-PCR amplification of characteristic fragments of the different splice variants.** The investigated sample genes with alternative splicing were *clpr2* **(A)**, *efg8* **(B)**, *hyd2* **(C)**, *lsg2* **(D)**, *mgmt* **(E)**, *nrnp1* **(F)**, *selEFf* **(G)**, *cyn23* **(H)**, *oee1* **(I)** and *ppi1* **(J)**. Primers were designed to amplify a characteristic fragment of each alternative splicing variant specifically (Figure 4 and Additional file 1: Table S3). The amplicons of the first (V1) and second (V2) alternative splicing variants have been cloned and sequenced. The expected lengths of fragments are given in Additional file 1: Table S3. DNA fragments that are consistent with the predictions are marked by arrowheads.

**Figure 6:**
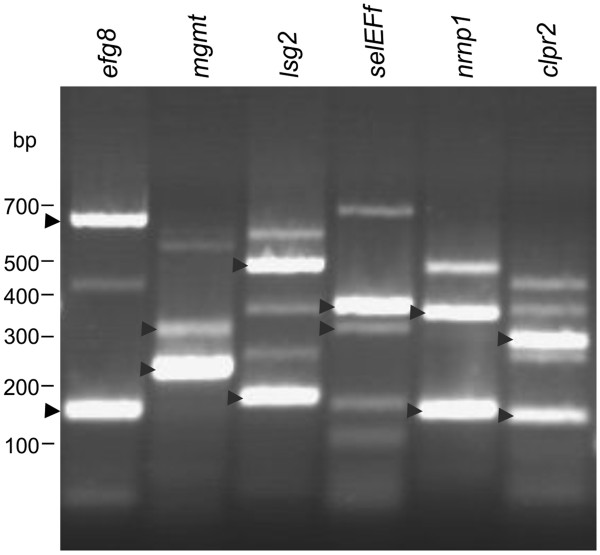
**Single-tube RT-PCR amplification of different splice variants.** The investigated sample genes with alternative splicing were *efg8*, *mgmt*, *lsg2*, *selEFf*, *nrnp1* and *clpr2*. Both splice variants of each gene were amplified in one and the same reaction using only a single pair of primers (Additional file 1: Table S3). The amplicons have been cloned and sequenced. The expected lengths of fragments are given in Additional file 1: Table S3. DNA fragments that are consistent with the predictions are marked by arrowheads.

**Figure 7:**
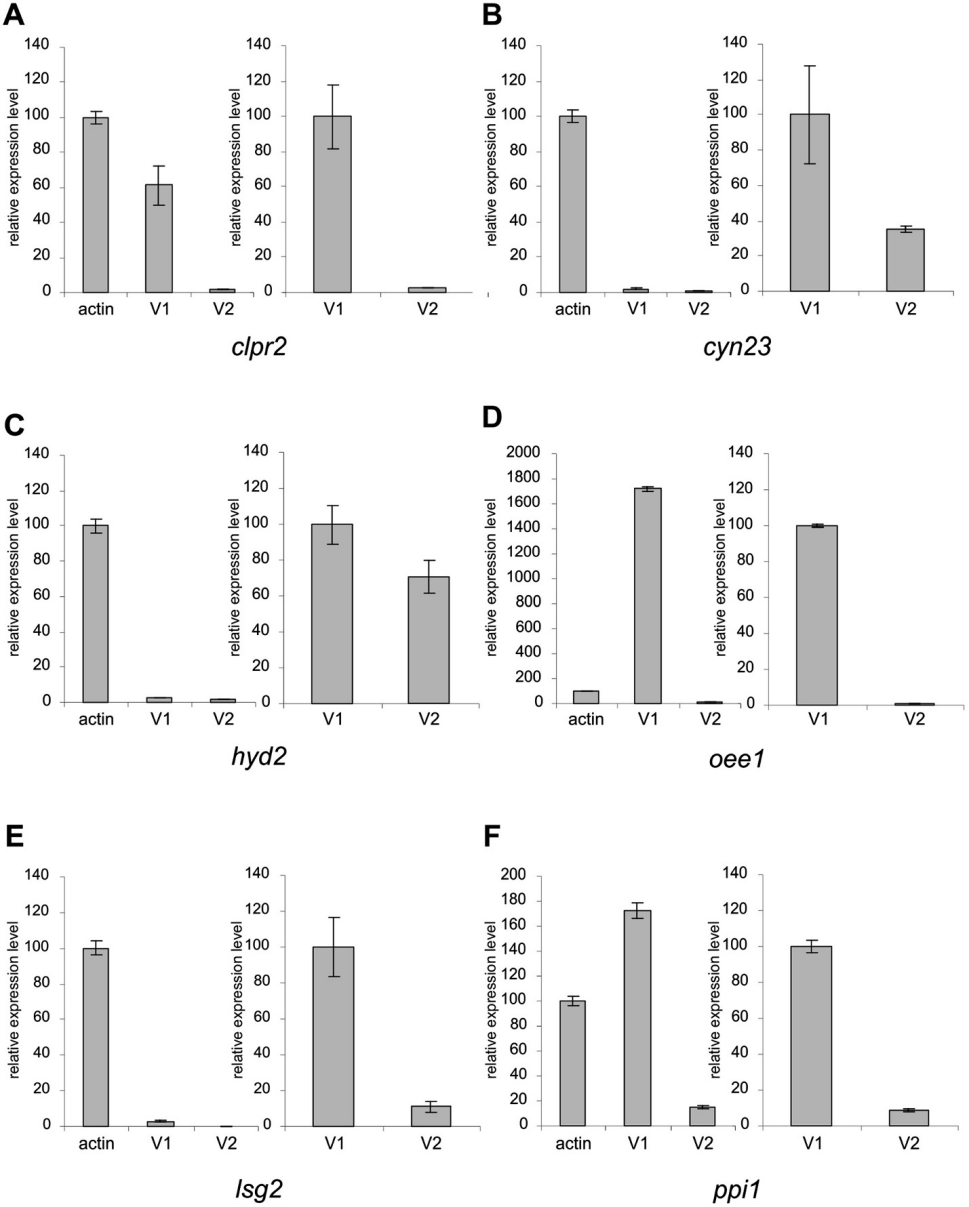
**Quantitative expression analysis of alternative splicing variants by real-time RT-PCR.** The investigated sample genes with alternative splicing were *clpr2* **(A)**, *cyn23* **(B)**, *hyd2* **(C)**, *oee1* **(D)**, *lsg2* **(E)** and *ppi1* **(F)**. Primers were designed to amplify a characteristic fragment of each alternative splicing variant specifically (Figure 4 and Additional file 1: Table S3). On the left side of each panel, the expression levels of both alternative splicing variants are shown in relation to the *actin* gene (the expression level of *actin* was set as 100%). On the right side of each panel, the expression levels of both alternative splicing variants are compared against each other (the expression level of V1 was set as 100%).

The first of the seven sample genes with exon skipping was *clpr2*. This gene codes for a highly conserved, ATP-dependent serine protease [69], which shows 86% identity to *Chlamydomonas* CLPR2 in an overlap of 285 amino acid residues [70]. Clp proteases are involved in many cellular and extracellular processes such as degradation of misfolded proteins, cell signaling and removing of short-lived regulatory proteins [69, 71]. The *clpr2* gene of *Volvox* is 2,599 bp in length (from start to stop codon) and includes 8 exons and 7 introns (Figure 4A, Additional file 1: Table S2). In the second splice variant of *clpr2*, exon five that is 135 bp in length is excluded from the mRNA by exon skipping (Figure 4A). The first splice variant encodes a 284-residue polypeptide whereas the second splicing variant encodes a 239-residue polypeptide (Additional file 1: Figure S1A, Additional file 1: Table S2). The structure of the ClpP protein isoform encoded by the second splice variant probably differs from the reported protein structure of ClpP [72], which is encoded by the first splice variant (Additional file 1: Figure S1B). The conserved amino acid residues F100, N111, Y119 and L120, which are involved in the α/β-type fold of the protein [72], are lacking in the second protein variant of *clpr2* (Additional file 1: Figure S1A). Both splice variants of *clpr2* have been amplified by RT-PCR to confirm the results of the bioinformatic analysis. For it, total RNA was isolated from synchronously growing female *Volvox* cultures at the stage of hatching. Pairs of primers were established to amplify each splice variant separately. For amplification of the first splice variant of *clpr2*, one primer, ON15266, resides on exon 5, which is lacking in the mRNA of the second splice variant; the second primer is ON15265 on exon 4 (Figure 4A and Additional file 1: Table S3). For verification of the second splice variant, one primer, ON15267, only binds to the exon-exon junction of exons 4 and 6, which emerges only after removal of a 659 bp fragment between exon 4 and exon 6 by splicing; the second primer was ON15265 on exon 4 (Figure 4A and Additional file 1: Table S3). A 134-bp cDNA fragment was predicted for variant 1 and a 117-bp fragment for variant 2; the RT-PCR yielded fragments of the expected sizes (Figure 5A, Additional file 1: Table S3). It was also possible to amplify both variants in one and the same reaction using only a single pair of primers (ON15265 and ON15371, Figure 4A and in Additional file 1: Table S3). Fragments of 278 bp (variant 1) and 143 bp (variant 2) were expected and actually obtained in the RT-PCR (Figure 6 and Additional file 1: Table S3); it should be mentioned that in addition to the correct fragments, some non-specific side products were amplified (Figure 6). Subsequently, the relative expression levels of both splice variants were measured by quantitative real-time RT-PCR, which is a sensitive method for analyzing relative expression levels of alternative splicing variants [49, 73–75]. As a reference gene for the quantitative real-time RT-PCRs the *actin* gene was used. The *actin* gene already has been used in previous studies as a reference in RT-PCR and quantitative real-time RT-PCR expression analyses [49, 76–78]. The primer pairs ON15265/ON15266 and ON15265/ON15267 were used to amplify the first and the second splice variant separately. Both primer pairs did not produce any non-specific fragments during RT-PCR reactions and the fragment sizes were between 100 and 200 bp which is the optimal fragment size for quantitative real-time RT-PCR [79]. The expression levels were calculated using the ∆∆Ct-method as described previously [78] and the results are shown in Figure 7A. The expression of the first splice variant of *clpr2* is ~1.6 fold less than *actin* and ~35.8 fold higher than the expression of the second splice variant (Figure 7A).

The second exon skipping candidate was *efg8* (Figure 4B, Additional file 1: Table S2). This gene codes for the mitochondrial elongation factor EF-TU and it is localized at the mating type locus of *Volvox* [50]. EF-TU elongation factors belong to the large family of GTP-binding elongation factors [80]. The first splice variant of *efg8* encodes a polypeptide of 453 amino acid residues, including three elongation factor domains, i.e. EF-TU, EFTU-II and EFTU-III (Additional file 1: Figures S2A-B). All three domains are essential for the elongation phase in protein synthesis [81]. The first domain, EF-TU, is the catalytic domain, which is responsible for the binding to the guanine nucleotide [82]. The non-catalytic domains II and III show a beta-barrel structure with six anti-parallel strands, which appear to be tightly associated to the catalytic domain [82, 83]. Interestingly, six exons are excluded in the second splice variant, which reduces the length of the mRNA by 510 nucleotides and the encoded polypeptide chain by 170 amino acid residues (Figure 4B, Additional file 1: Figure S2A). In the second variant, 139 amino acid residues of the catalytic EF-TU domain are lacking, which will in all likelihood affect the binding properties of the domain. Moreover, GTP-binding proteins usually have two conserved sequences with the consensus sequences Asn-Lys-x-Asp (residues 192 to 195 in the first variant) and Ser-Ala-Leu/Lys (residues 230 to 233 in the first variant), which are important for the binding to a guanine nucleotid [84]; both motives are lacking in the second variant (Additional file 1: Figure S2A). The first part of the EFTU-II domain also is absent in the second variant, which should cause altered binding properties and, thus, affect its activity in RNA translation [85, 86]. For amplification of the first splice variant of *efg8* by RT-PCR, the primer ON15273 resides on exon 5 and primer ON15274 is located on exon 6; both exons are lacking in the mRNA of the second splice variant. For verification of the second splice variant, one primer, ON15271, only binds to the exon-exon junction of exons 4 and 11, which emerges only after removal of a large fragment (10772 bp) between exon 4 and exon 11 by splicing; the second primer was ON15272 on exon 12. A 151-bp cDNA fragment was predicted for variant 1 and a 176-bp fragment for variant 2; the RT-PCR yielded fragments of the expected sizes (Figure 5B, Additional file 1: Table S3). It was also possible to amplify both variants in one and the same reaction using only a single pair of primers (ON15365 and ON15366, Figure 4B and in Additional file 1: Table S3). Fragments of 664 bp (variant 1) and 154 bp (variant 2) were expected and actually obtained in the RT-PCR (Figure 6 and Additional file 1: Table S3). The relative expression levels of both splice variants of *efg8* could not be determined by quantitative real-time RT-PCR because non-specific side products defeated the analysis repeatedly (the same was true for *mgmt*, *nrnp1* and *selEFf*).

The third sample gene was *hyd2*. This gene codes for an iron hydrogenase, which catalyzes the reversible conversion of molecular hydrogen to protons and electrons [87, 88] (Figure 4C, Additional file 1: Table S2). The first splice variant of *hyd2* encodes a polypeptide with a large Fe-only hydrogenase domain, which is 352 amino acid residues long (residues 82 to 434). In the second splice variant, 169 amino acid residues are lacking at the N-terminal end of the polypeptide (Figure 4C, Additional file 1: Figure S3A), including 88 amino acid residues of the Fe-only hydrogenase domain [89, 90]. The X-ray crystal structure of the Fe-only hydrogenase from *Clostridium pasteurianum* could show that the amino acid residues 90 to 97 and 130 to 135 are essential to form β sheets around the active site [91] (Additional file 1: Figure S3A). Lack of this part in the second splice variant should change the protein structure and, as a consequence, the enzyme characteristics. In contrast, the actual active site is present in both variants of the Fe-only hydrogenase [90] (Additional file 1: Figure S3A). There are three conserved protein sequence motifs in the active site of variant 1, motif 1 (PMFTSCCPxW, residues 169 to 178), motif 2 (MPCxxKxxExxR, residues 228 to 239) and motif 3 (FxExMACxGxCV, residues 415 to 426), and variant 2 contains exactly the same motifs except for the very first amino acid residue of motif 1. For amplification of the first splice variant of *hyd2* by RT-PCR, one primer, ON15276, resides on exon 3, which is lacking in the mRNA of the second splice variant; the second primer was ON15276 on exon 1 (Figure 4C and Additional file 1: Table S3). For verification of the second splice variant, one primer, ON15277, only binds to the exon-exon junction of exons 2 and 5, which emerges only after removal of a 818 bp fragment between exon 2 and exon 5 by splicing; the second primer was ON15275 on exon 1 (Figure 4C and Additional file 1: Table S3). A 128-bp cDNA fragment was predicted for variant 1 and a 102-bp fragment for variant 2; the RT-PCR yielded fragments of the expected sizes (Figure 5C, Additional file 1: Table S3); the band of variant 2 showed a lower intensity than the band of variant 1. It was not possible to amplify both variants of *hyd2* in one and the same reaction, instead only one of both variants was amplified (the same was true for *cyn23*, *oee1* and *ppi1*). However, the relative expression levels of both splice variants could be determined by quantitative real-time RT-PCR. The expression of the first splice variant of *hyd2* is only ~1.5 fold higher than the expression of the second splice variant (Figure 7C). Compared to *actin*, both splice variants are expressed at a very low level, i.e. *hyd2* variants 1 and 2 account for only 2.4% and 1.7%, respectively, of the *actin* expression level (Figure 7C).

The fourth exon skipping candidate was *lsg2*. This *Volvox* gene codes for a matrix metalloproteinase; *lsg2* was shown to be expressed with an above-average rate during the late developmental stages in somatic cells [92] (Additional file 1: Table S2). Lsg2 (variant 1) shows 31% identity to the gamete lytic enzyme (GLE) of *Chlamydomonas* in an overlap of 537 amino acid residues. GLE of *Chlamydomonas is* a proteinase, which degrades cell walls of gametes during mating [93]. For enzymatic degradation, it contains a large peptidase M11 domain (amino acid residues 146 to 458 in variant 1, Additional file 1: Figure S4A) The M11 domain is conserved among several metalloproteinases including VMPs [94, 95]. The typical HExxHxxGxxH motif, which contains four histidine residues for zinc binding in the active site of the enzyme [96] can be found in variant 1 of Lsg2 (amino acid residues 301 to 312, Additional file 1: Figure S4A). Another motif, which is believed to be responsible for the binding to calcium, is also conserved in variant 1 of Lsg2 (amino acid residues 500 to 512, Additional file 1: Figure S4A). In contrast, 103 amino acid residues of the peptidase M11 domain are lacking in the polypeptide encoded by the second splice variant, because exon nine (309 bp) is excluded by exon skipping (Figure 4D, Additional file 1: Figure S4A). For amplification of the first splice variant of *lsg2* by RT-PCR, one primer, ON15295, resides on exon 9, which is lacking in the mRNA of the second splice variant; the second primer was ON15294 on exon 8 (Figure 4D and Additional file 1: Table S3). For verification of the second splice variant, one primer, ON15296, only binds to the exon-exon junction of exons 8 and 10, which emerges only after removal of a 2285 bp fragment between exon 8 and exon 10 by splicing; the second primer was ON15294 on exon 8 (Figure 4D and Additional file 1: Table S3). A 137-bp cDNA fragment was predicted for variant 1 and a 131-bp fragment for variant 2; the RT-PCR yielded fragments of the expected sizes (Figure 5D, Additional file 1: Table S3). It was also possible to amplify both variants in one and the same reaction using only a single pair of primers (ON15294 and ON15368, Figure 4D and Additional file 1: Table S3). Fragments of 482 bp (variant 1) and 173 bp (variant 2) were expected and actually obtained in the RT-PCR (Figure 6 and Additional file 1: Table S3); it should be mentioned that in addition to the correct fragments, some non-specific side products were amplified (Figure 6). Quantitative real-time RT-PCR showed that both splice variants are expressed at a very low level in comparison to *actin*. However, the expression of the first splice variant of *lsg2* is ~10 fold higher than the expression of the second splice variant (Figure 7E).

The fifth sample gene was *mgmt*. This gene codes for a putative 6-O-methylguanine DNA methyltransferase (Additional file 1: Table S2). The O-6-methylguanine-DNA methyltransferase is essential for viability because it reverses DNA alkylation damage by removing the offending alkyl group [97–99]. The first splice variant of *mgmt* encodes a polypeptide with 153 amino acid residues in length (Additional file 1: Table S2, Additional file 1: Figure S5A) and contains a DNA binding domain, which is 89 amino acid residues long [100] (Additional file 1: Figure S5A,B). As a result of the exon skipping event, 29 residues are lacking in the DNA binding domain of the second variant (Figure 4E, Additional file 1: Figure S5A). Some of these 29 amino acid residues were previously shown to be involved in DNA binding [101]. For example, the tyrosine residue at position 44 of variant 1 (Y44, Additional file 1: Figure S5A) has been shown to be a key residue involved in recognition of the O6-alkylguanine lesion through a hydrogen bond with the N3 atom of the modified base [101, 102]. Furthermore, the arginine residue at position 56 of variant 1 (R56, Additional file 1: Figure S5A) is necessary for the repair of base damage in duplex DNA [100]. The absence of these two amino acid residues most probably affects the binding characteristics of the second protein variant. For amplification of the first splice variant of *mgmt* by RT-PCR, one primer, ON15279, resides on exon 2, which is lacking in the mRNA of the second splice variant; the second primer was ON15278 on exon 1 (Figure 4E and Additional file 1: Table S3). For verification of the second splice variant, one primer, ON15280, only binds to the exon-exon junction of exons 1 and 3, which emerges only after removal of a 276 bp fragment between exon 1 and exon 3 by splicing; the second primer was ON15278 on exon 1 (Figure 4E and Additional file 1: Table S3). A 173-bp cDNA fragment was predicted for variant 1 and a 165-bp fragment for variant 2; the RT-PCR yielded fragments of the expected sizes (Figure 5E, Additional file 1: Table S3); in the PCR of variant 1 also two larger, non-specific side products were amplified, as verified by cloning and sequencing. It was also possible to amplify both variants in one and the same reaction using only a single pair of primers (ON15278 and ON15367, Figure 4E and Additional file 1: Table S3). Fragments of 310 bp (variant 1) and 223 bp (variant 2) were expected and actually obtained in the RT-PCR (Figure 6 and Additional file 1: Table S3); it should be mentioned that in addition to the correct fragments, a larger, non-specific side product was amplified (Figure 6).

The sixth exon skipping candidate was *nrnp1*. This *Volvox* gene codes for a polypeptide with two RNA recognition motif (RRM) domains, also known as RNA binding domains (RBDs). The RRM domain is by far the most abundant type of eukaryotic RNA-binding motif. This domain is involved in different cellular processes like mRNA and rRNA processing, RNA export and RNA stability [103–105]. The first splice variant of *nrnp1* encodes a polypeptide with 344 amino acid residues; the RRMs are localized at amino acid residues 76 to 132 and 150 to 219 (Additional file 1: Table S2, Additional file 1: Figure S6A). The protein product of the second splice variant is 179 amino acid residues shorter than the first variant (Figure 4F and Additional file 1: Figure S6). In the second variant, the first RRM domain is lacking and the second RRM domain is truncated, i.e., 29 amino acid residues are lacking (Additional file 1: Figure S6A). The crystal structure analysis of the RRM domain previously showed that the first part of the domain is important for correct folding [106]. The complete elimination of the first RRM and the truncation of the second RRM domain most probably change the RNA binding capacity significantly in the second variant. For amplification of the first splice variant of *nrnp1* by RT-PCR, one primer, ON15287, resides on exon 2, which is lacking in the mRNA of the second splice variant; the second primer was ON15286 on exon 1 (Figure 4F and Additional file 1: Table S3). For verification of the second splice variant, one primer, ON15288, only binds to the exon-exon junction of exons 1 and 3, which emerges only after removal of a 542 bp fragment between exon 1 and exon 3 by splicing; the second primer was ON15286 on exon 1 (Figure 4F and Additional file 1: Table S3). A 116-bp cDNA fragment was predicted for variant 1 and a 107-bp fragment for variant 2; the RT-PCR yielded fragments of the expected sizes (Figure 5F, Additional file 1: Table S3). It was also possible to amplify both variants in one and the same reaction using only a single pair of primers (ON15286 and ON15370, Figure 4F and Additional file 1: Table S3). Fragments of 342 bp (variant 1) and 152 bp (variant 2) were expected and actually obtained in the RT-PCR (Figure 6 and Additional file 1: Table S3); it should be mentioned that in addition to the correct fragments, a larger, non-specific side product was amplified (Figure 6).

The seventh and last exon skipping candidate was *selEFf*, which is localized at the mating type locus of *Volvox* [50] (Additional file 1: Table S2). This gene codes for a putative selenocysteine-specific elongation factor (selEFf). Such translation factors are necessary for the incorporation of selenocysteine into proteins; selEFfs probably replace EF-Tu for the insertion of selenocysteine directed by the UGA codon [107]. In the first splice variant of *selEFf*, a very short exon (exon 4, 56 bp) is flanked by two very large introns, 7,996 bp and 8,313 bp in length (Figure 4G). This exon is excluded from the second splice variant by exon skipping and thus, an intron of 16,365 bp is spliced out. This intron seems to be the largest intron reported so far in *Volvox*. The alternative splicing event in the second variant also introduces a premature stop codon into the open reading frame (Additional file 1: Figure S7A). Unfortunately, no information about the structure of selEFf proteins is available, but the elimination of 142 amino acid residues in the second splice variant means that the length of the polypeptide is almost halved relative to the first variant and this significant cut-off probably affects the structure and characteristics of this translation factor. For amplification of the first splice variant of *selEFf* by RT-PCR, one primer, ON15290, resides on exon 4, which is lacking in the mRNA of the second splice variant; the second primer was ON15289 on exon 3 (Figure 4G and Additional file 1: Table S3). For verification of the second splice variant, one primer, ON15291, only binds to the exon-exon junction of exons 3 and 5, which emerges only after removal of a 16365 bp fragment between exon 3 and exon 5 by splicing; the second primer was ON15289 on exon 3 (Figure 4G and Additional file 1: Table S3). A 196-bp cDNA fragment was predicted for variant 1 and a 166-bp fragment for variant 2; the RT-PCR yielded fragments of the expected sizes (Figure 5G, Additional file 1: Table S3); in addition to the correct fragments, a larger, non-specific side product was amplified in the RT-PCR for the second variant. It was also possible to amplify both variants in one and the same reaction using only a single pair of primers (ON15289 and ON15369, Figure 4G and Additional file 1: Table S3). Fragments of 357 bp (variant 1) and 301 bp (variant 2) were expected and actually obtained in the RT-PCR (Figure 6 and Additional file 1: Table S3); in addition to the correct fragments, some non-specific side products were amplified (Figure 6).

#### Verification of mutually exclusive exons

The sample gene for mutually exclusive exons was *cyn23*, which encodes a cyclophilin-related protein (Additional file 1: Table S2). Cyclophilins are ubiquitous proteins that belong to the family of peptidyl-prolyl cis/trans isomerases (PPIases) [108], also known as immunophilins. These immunophilins are proposed to function in protein folding, protein degradation, stress response, signal transduction and pre-mRNA splicing [109–113]. The *cyn23* gene contains two alternate mutually exclusive exons, exons 4 and 5. Because both exons are 106 nucleotides in length (Figure 4H, Additional file 1: Figure S11), the number of amino acid residues of the two protein isoforms is identical. However, the two isoforms differ from each other at 13 amino acid positions: A125G, G127Y, G128D, N129D, K130P, G132S, A133G, R134A, V139I, E150Q, A152T, I153A and G155A (Additional file 1: Figure S8A). Some of these residues, like alanine at position 125, have been reported to be part of the cyclosporin-binding site [114, 115]. Moreover, exchange of amino acids between positions 116 and 155 by site-directed mutagenesis was shown to affect the binding properties of cyclophilins [116, 117]. Therefore, the differences in amino acid sequence between the two isoforms might produce isoforms with different binding properties. For amplification of the first splice variant of *cyn23* by RT-PCR, one primer, ON15269, resides on exon 4 and the second primer is ON15268 on exon 3 (Figure 4H and Additional file 1: Table S3). For verification of the second splice variant, one primer, ON15270, resides on exon 5 and the second primer again was ON15268 on exon 3. A 145-bp cDNA fragment was predicted for variant 1 and a 146-bp fragment for variant 2; the RT-PCR yielded fragments of the expected sizes (Figure 5H, Additional file 1: Table S3). Due to the almost identical fragment sizes and the resulting identical migration distances in the gel, both variants were not amplified in one and the same reaction. Quantitative real-time RT-PCR showed that both splice variants are expressed at a very low level in comparison to *actin*. However, the expression of the first splice variant of *cyn23* is ~2.8 fold higher than the expression of the second splice variant (Figure 7B).

#### Verification of intron retention

The candidate gene for verification of intron retention was *oee1*, which codes for a subunit of the oxygen evolving complex of photosystem II (Additional file 1: Table S2). Previously, it was demonstrated that *oee1* is subject to germline-specific expression in *Volvox* [118, 119]. The first splice variant of *oee1* encodes a polypeptide of 297 amino acid residues (Additional file 1: Figure S9A, Additional file 1: Table S2), which shows 87% identity to OEE1 of *Chlamydomonas* in an overlap of 294 amino acid residues [120]. It includes a large manganese-stabilizing protein (MSP) domain (Additional file 1: Figure S9A, B), which is required for photosystem II assembly, stability and photoautotrophy [121]. In the second splice variant of *oee1*, the first intron (69 bp in length) is retained by alternative intron retention (Figure 4I), which potentially leads to an N-terminally truncated protein isoform with a shortened MPS domain (Additional file 1: Figure S9A,B).

For verification of the first splice variant of *oee1* by RT-PCR, one primer, ON15285, only binds to the exon-exon junction of exons 1 and 2, which emerges only after removal of intron 1 (69 bp) by splicing; the second primer was ON15281 on exon 1 (Figure 4I and Additional file 1: Table S3). For amplification of the second splice variant, one primer, ON15284, resides on the retained intron sequence (intron 1 of variant 1), which therefore is lacking in the mRNA of the first splice variant; the second primer was ON15281 on exon 1 (Figure 4I and Additional file 1: Table S3). A 111-bp cDNA fragment was predicted for variant 1 and a 158-bp fragment for variant 2; the RT-PCR yielded fragments of the expected sizes (Figure 5I, Additional file 1: Table S3). Quantitative real-time RT-PCR showed that the expression of the first splice variant of *oee1* is ~17 fold higher than *actin*; whereas the expression of the second variant is ~11 fold less than *actin* (Figure 7D).

The bioinformatic analysis of EST sequences revealed a third splice variant of *ooe1*. For verification of this variant by RT-PCR, one primer, ON15283, only binds to the exon-exon junction of exons 1 and 3, which emerges only after removal of a 356 bp fragment between exon 1 and exon 3 by splicing; the second primer was ON15281 on exon 1 (Figure 4I and Additional file 1: Table S3). A 112-bp cDNA fragment was predicted for variant 3. However, we were not able to confirm this variant by RT-PCR (Figure 5I, middle lane).

#### Verification of alternative 5′ splice sites

The sample gene for alternative 5′ splice sites was *ppi1*, which codes for a protein with an Ypi1 domain (Additional file 1: Table S2). The Ypi1 domain is a *Saccharomyces cerevisiae* type 1 protein phosphatase inhibitor [122]. The gene *ppi1* of *Volvox* is a quite small gene with a single intron, which is 100 bp in length. The first splice variant of *ppi1* encodes a 100-amino-acid polypeptide, which shows 82% identity to the FAP255 protein of *Chlamydomonas*. FAP255 is a flagellar associated protein found in the flagellar proteome [59, 123] (Additional file 1: Table S2, Additional file 1: Figure S10A). In the second splice variant, 21 bp at the 5′ side of the intron are retained and thereby a premature stop codon is introduced (Figure 4J). As a consequence, a shortened protein isoform with a truncated Ypi1 domain is produced (Additional file 1: Figure S10B).

For verification of the first splice variant of *ppi1* by RT-PCR, one primer, ON15302, only binds to the exon-exon junction of exons 1 and 2, which emerges only after complete removal of the 100 bp of intron 1 by splicing; the second primer is ON15300 on exon 1 (Figure 4J and Additional file 1: Table S3). For amplification of the second splice variant, one primer, ON15301, resides on the retained 21 bp of intron 1, which therefore is lacking in the mRNA of the first splice variant; the second primer was ON15300 on exon 1 (Figure 4J and Additional file 1: Table S3). A 140-bp cDNA fragment was predicted for variant 1 and a 141-bp fragment for variant 2; the RT-PCR yielded fragments of the expected sizes (Figure 5J, Additional file 1: Table S3); the band of variant 2 showed a lower intensity than the band of variant 1. Quantitative real-time RT-PCR showed that the expression of the first splice variant of *ppi1* is ~70% higher than the expression of *actin* and even ~1000% higher than the expression of variant 2.

## Discussion

### A comparative view on alternative splicing in *Volvox*

This work provides a bioinformatic analysis of 132,038 ESTs in relation to alternative splicing events in the multicellular green alga *Volvox*. The results show that 66.7% of the alternative splice events are within the coding region (Figure 3A) and thus have an effect on the protein sequence and, as a consequence, frequently also on protein structure and function. The remaining 33.3% of all alternative splice events in *Volvox* were within the 5′ and 3′ UTRs (Figure 3A), which is higher than reported data (21-28.5%) from *Arabidopsis* (Figure 3C), mouse and human [5, 124–127] and this study). Thus, UTRs in the alga *Volvox* are more frequently target of alternative splicing than UTRs in the land plant *Arabidopsis* (Figures 3A and 3C). Alternative splicing of UTRs can play a key role both in regulation and in the production of mRNA diversity [11, 128]. Moreover, changes in mRNA secondary structure can affect RNA processing, mRNA stability and translation of the messenger [129–131]. In addition, it possibly creates phenotypic variability [132]. In multicellular organisms, regulation of mRNA stability plays a crucial role in development, growth and differentiation [133]. That data indicate that there might be more variability and diversity through alternative splicing in *Volvox* UTRs than in *Arabidopsis* UTRs (Table 1, Figures 3A and 3C).

**Table 1:** **The occurrence of alternative splicing events in** ***Volvox*** **in comparison to** ***Chlamydomonas*** **,** ***Arabidopsis*** **and human**

Organism	Total number of included ESTs/cDNAs	Alternative splicing [%]	Exon skipping [%]	Intron retention [%]	Alternative 5′ splice site [%]	Alternative 3′splice site [%]
*Volvox carteri* ^1*^	132,038	2.9	9.5	46.5	17.9	21.9
*Chlamydomonas reinhardtii* ^2**^	252,484	3	11.9	50.0	11.6	25.8
*Arabidopsis thaliana* ^3^	541.594	20	3	41	18	38
Human^4***^	435 million cDNA reads	90	40	3	8	18

^1^ this study, ^2^ [53], ^3^ [26],^4^ [16, 134].

*4.2% show other alternative splicing events.

**0.7% show both alternative 5′ splice site and alternative 3′ splice sites.

*** 32.7 % show other alternative splicing events such as exclusive exon, alternative first exon, alternative last exon and tandem 3′ UTR.

The distribution scheme of the different types of alternative splicing in *Volvox* shows that intron retention is the predominant type, while exon skipping is only a smaller part (46.5% versus 9.5%; Table 1, Figure 2A). Interestingly, the situation in human is just opposite to the situation in *Volvox*: there are only 1% intron retention events and 35.6% exon skipping events (Table 1). These highly differing distributions could result from different genome features. For example, the introns in human are much longer than in *Volvox*, or to be more exact, the median sizes of introns are 1,504 bp versus 358 bp [58, 135]. The size of introns is crucial factor in the mechanism of intron retention, i.e., short introns were shown to be much more frequently retained than longer introns [136–138]. On the contrary, an increase in intron length correlates positively with promotion of the exon skipping mechanism [65, 139, 140]. Therefore, intron length is the determining factor for the switch from the intron definition mechanism, in which the 5′ and 3′ splice sites are initially recognized and paired across the intron, to the exon definition mechanism, in which splice sites are paired first across the exons, with spliceosome assembly proceeding through subsequent pairing of exon units [141]. Thus, a plethora of short introns is *Volvox* is probably recognized and spliced out through the intron definition mechanism, while in human the exon definition mechanism is dominant because a vast number of exons is flanked by long introns [140, 141]. It should be noted that the introns of *Chlamydomonas* are even somewhat (~25%) shorter than those in *Volvox*, i.e., the average lengths are 491 bp and 373 bp, respectively (Additional file 1: Table S1), but the calculated intron retention rate is about the same in both organisms (Figure 2, Table 1). However, the intron lengths of both species can be considered as short and therefore the intron definition mechanism seems to apply for both species to the same extend.

Our analysis of ESTs in *Volvox* revealed that 2.9% of all *Volvox* genes undergo alternative splicing, which is similar to the reported 3% for *Chlamydomonas* [53]. The absolute numbers of alternative splicing events were 580 for *Volvox* and 493 for *Chlamydomonas* (Figure 2). However, it should be noted that the absolute numbers and percentages have been calculated based on different total EST numbers, i.e., the number of analyzed ESTs in *Chlamydomonas* (252,484) was almost twice that of *Volvox* (132,038) (Table 1) even if the total number of protein-coding loci is about the same in *Volvox* (14,520) and *Chlamydomonas* (14,516) [58]. Because the total number of ESTs is a critical value for detection of alternative splicing events, probably more alternative splicing events remained undetected in *Volvox* than in *Chlamydomonas.* These results and considerations indicate that actually the rate of alternatively spliced genes is higher in *Volvox* than in *Chlamydomonas* and, thus, also the variability and diversity through alternative splicing appears to be higher in *Volvox*. However, the data presented here are not corrected for the amount of transcript evidence available.

The number of ESTs analyzed in *Volvox* (132,038) is similar to the number of ESTs analyzed in *Arabidopsis* (176,915) in a study by Zhu et al. in 2003 [22], which came to a value of 1.5% alternatively spliced genes for *Arabidopsis* (Additional file 1: Figure S12). As mentioned above, nine years later, after sequencing countless more ESTs, the rate was calculated to be 61% [18, 19]. Therefore, in actual fact, alternative splicing in the alga *Volvox* might be just as common as in higher eukaryotes like *Arabidopsis*, *Drosophila* or even human [13, 17] [14–16, 19].

### Some striking alternative splicing events in *Volvox*

During our genome-wide analysis, we found some genes with remarkable alternative splicing variants regarding the size or number of excluded introns and exons.

One of these genes is *efg8*, which is subject to exon skipping (Figure 4B). The first splicing variant of *efg8* contains 15 exons. In the second splicing variant even six consecutive exons (5 to 10) are skipped at once. These exons are quite short, i.e., between 44 and 121 bp in size, and also the sequences between the exons are short (between 162 and 718 bp). However, two extremely long introns flank the skipped sequence: The introns 4 (between exon 4 and 5) and 10 (between exon 10 and 11) are 3,958 and 4,407 bp in length, respectively (Figure 4B). Thus, in the second splicing variant an intron of 10,772 bp is spliced out, which contains exons 5-10 and introns 4-11. This spliced fragment of the second variant is one of the longest introns identified in *Volvox* so far (Figure 4B). In *Arabidopsis* and maize, the longest reported introns are about 3 and 7 kb in length, respectively [142–144]. The longest introns among all plants have been identified in tobacco and rice, which are about 17 and 28 kb in length, respectively [145, 146]. In general, especially long introns can contain regulatory elements to control gene expression under certain conditions (development, cell-type specificity, environmental influences) [147, 148].

The *selEFf* gene is also subject to exon skipping. The first splicing variant of *selEFf* contains 7 exons. In the second splicing variant a single small exon (exon 4), only 56 bp in length, is skipped. This exon is flanked by two extremely long introns, which are 7,996 and 8,313 bp in length, respectively (Figure 4G). Thus, in the second splicing variant an intron of 16,365 bp is spliced out (Figure 4G), which contains exon 4, intron 3 and intron 4. This spliced ~16 kb fragment of the second variant is one of the longest introns identified in the plant lineage so far; it is longer than any intron in *Arabidopsis* or maize.

### Alternative splicing and organismal complexity

Alternative splicing is a major mechanism for generating proteome diversity, which probably was co-opted in evolution of multicellular complexity [149]; it plays a critical role in differentiation and development of multicellular eukaryotes [128, 150]. Because *Volvox* only has about the same number of protein-coding genes compared to its unicellular relative *Chlamydomonas*, alternative splicing could have contributed to an increase in proteome diversity when a unicellular *Chlamydomonas*-like ancestor evolved to the multicellular *Volvox* alga with its differentiated cells. In more concrete terms, the transition to multicellularity in volvocine algae required a proteome with an increased capacity to address new traits and tasks like multicellularity, cell differentiation, multicellular motility and phototaxis as well as egg and sperm formation. Alternative splicing seems to be particularly important for the generation of new or modified regulatory proteins, which are indispensable in the evolution of multicellular complexity [125]. Especially transcription factors and signal transducers that act as key regulators in complex, multicellular systems seem to be subject to extensive alternative splicing [124, 125, 151].

**Figure 8:**
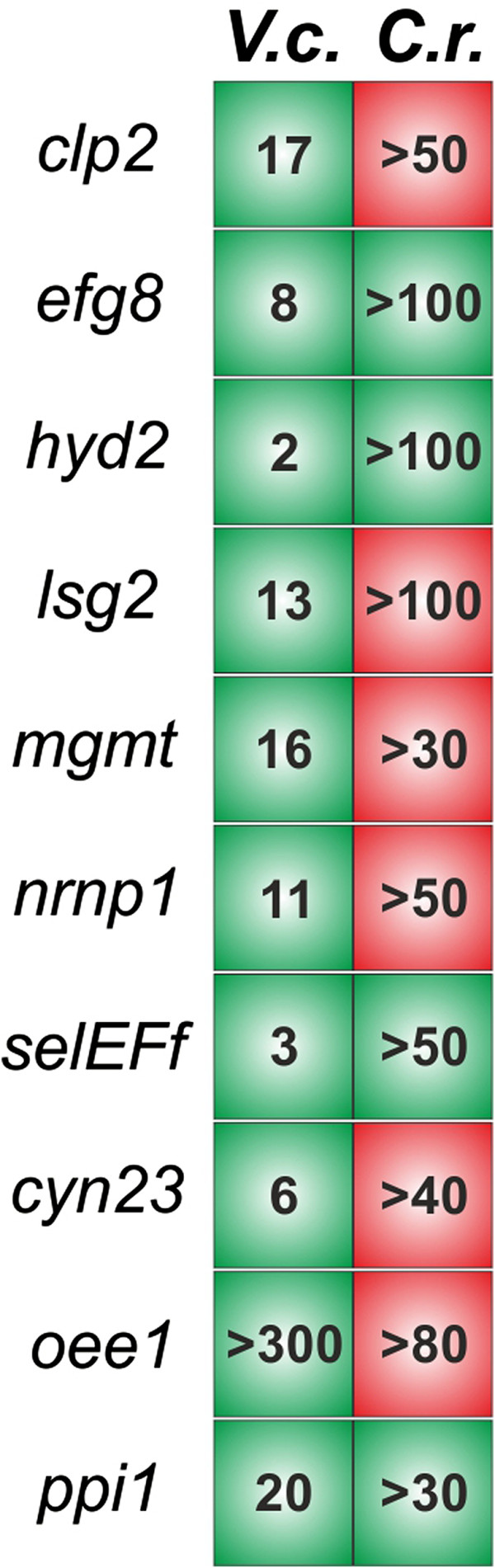
**Alternative splicing status of orthologous gene pairs of** ***Volvox*** **and its closely related, unicellular relative** ***Chlamydomonas*** **.** Comparison of the ten investigated sample genes of *Volvox* with their orthologs in *Chlamydomonas* regarding alternative splicing events. A green background shows genes with alternative splice events, a red background indicates that genes do not exhibit alternative splicing. Numbers indicate the EST coverage of given genes in *Volvox* (*V.c.*) or *Chlamydomonas* (*C.r.*).

In our analysis we found some evidence that alternative splicing actually affects key regulators. For example, the retinoblastoma gene *rbr1/mat3*, which codes for a key cell cycle and cell size regulator [49, 152], is only subject to alternative splicing in the multicellular alga *Volvox*; no such event has been reported its close unicellular relative *Chlamydomonas* [49, 50, 152]. This gene also shows gender-specific splicing in *Volvox* and it is believed to be involved by the evolution of oogamy [50, 153]. Also worth mentioning is the general distribution of alternatively spliced genes in *Volvox* in relation to its unicellular relative *Chlamydomonas.* For it, we took one-hundred alternatively spliced genes from *Volvox* and compared them with the corresponding orthologs in *Chlamydomonas.* Ten of these one-hundred genes were those that were selected for experimental verification and another ninety alternatively spliced genes were randomly selected. The results are shown in Figure 8 and Additional file 1: Table S4. The comparison demonstrates that the largest fraction of alternatively spliced genes in *Volvox* (70% of the analyzed genes) does not exhibit alternative splicing in the unicellular alga *Chlamydomonas*. The lack of alternative splicing events for those genes in *Chlamydomonas* is not due to poor EST support, because the total number of ESTs is lower for the *Volvox* genes than for their orthologs in *Chlamydomonas* (Figure 8 and Additional file 1: Table S4)*.* This observation supports the idea that alternative splicing events increased during evolution from the unicellular ancestor to the multicellular alga *Volvox* in order to expand the transcript diversity and, as a consequence, the proteomic diversity. Recently, the group of Urrutia also demonstrated a strong association between alternative splicing and organism complexity [1]. In addition to alternative splicing, gene duplication followed by divergence also increases proteome diversity [65]. During evolution of multicellular volvocine algae, the number of members in several protein families increased significantly through gene duplication. The families with the most extensive expansions are the VMPs, the pherophorins and the cyclin Ds, which are involved in the (partly gender-specific) biosynthesis of the extracellular matrix and in the regulation of cell division program during development [58].

However, in contrast to the extent of gene duplications, which remains invariant after sequencing of the genome, it can be expected that the percentage of alternatively spliced genes will increase strongly in *Volvox* when more and more EST data and full-length cDNA sequences become available, just as it happened earlier in *Arabidopsis* [18, 19, 22–24, 154]. In all probability, deep sequencing of the *Volvox* transcriptome during asexual and sexual development, during embryonic cleavage divisions and cellular differentiation and under various environmental conditions (e.g., heat and light stress or nutrient deprivation) will yield numerous new splicing variants. Thus, it is becoming increasingly clear that alternative splicing is not an exception, but relatively widespread in *Volvox* and other eukaryotes. This degree raises quite difficult questions: Do all splice isoforms have functional significance? How big is the noise in the splicing process? At which extent do truncated and misfolded proteins play a role in cellular regulation? Which amount of inadvertently produced isoforms can be tolerated by a cell? However, frequently alternative splicing seems to be part of molecular mechanisms that allow (some) cells of an organism to decrease the concentration of certain (functional) proteins without changing the transcription rate of the corresponding genes. Instead, shortened or modified non-functional mRNA variants are generated by alternative splicing. In this way, specific cellular and physiological processes can be attenuated, altered or even intensified at the post-transcriptional level.

In human, splicing error rates of 1 to 10 percent have been calculated [155, 156], but it remains difficult to distinguish between functional and non-functional isoforms because also truncated proteins might have a function. In several studies, criteria like abundance, conservation of splicing events across species, tissue specificity and developmental stage specificity have been used to assess functionality [155, 157]. Different models proceed on the assumption that the splicing machinery makes mistakes at a constant error rate, is dependent on the number of introns or is determined by the number of introns and transcript abundance [155, 157].

However, the noise in the splicing process should not be considered as a collateral damage of splicing. Noise in splicing might have been an important factor in the evolution of multicellularity in volvocine algae because it creates a landscape of opportunities in which novel biological activity can be explored at very little cost [155].

## Conclusions

Our results show that the approach for prediction of alternative splicing events in *Volvox* was accurate and reliable. Moreover, quantitative real-time RT-PCR appears to be useful for analyses of relationships between the appearance of specific alternative splicing variants and different kinds of physiological, metabolic and developmental processes as well as responses to environmental changes.

## Methods

### Strain and culture conditions

Synchronous cultures of *Volvox carteri* f. *nagariensis* strain EVE (wild-type female) [158] were grown in standard *Volvox* medium [159] at 28°C in an 8 h dark/16 h light (10,000 lux) cycle [160].

### Data sources

Genomic sequences of *Volvox carteri* f. *nagariensis* (strain Eve) came from the *Volvox carteri* whole genome shotgun sequencing project [58] (GenBank project accession No. ACJH00000000). The genomic contigs of *Volvox* are available both on the web pages of phytozome (v10), the plant comparative genomics portal of the Department of Energy’s Joint Genome Institute (JGI) (http://www.phytozome.net/), and on the web pages of the *Volvox* genome portal of JGI (http://genome.jgi-psf.org/Volca1/Volca1.info.html). The sequences of 132,038 ESTs of *Volvox carteri* f. *nagariensis* were generated as part of the *Volvox* genome project [58] and are available at the GenBank EST database (http://www.ncbi.nlm.nih.gov/dbEST/) and at the PlantGDB web resource (http://www.plantgdb.org/). These ESTs were derived from the female *Volvox carteri* f. *nagariensis* strains Eve and Eve10 and the male strain 69-1b [58]. RNAs from Eve and 69-1b were isolated 1.5, 10, 24, 48 hours after sexual-induction and subsequently pooled. For Eve10, extracted RNAs from 2-4 and 32-128 cell stages were pooled. The sequences both of genomic contigs and ESTs of *Chlamydomonas* came from the *Chlamydomonas* whole genome shotgun sequencing project [59]. The data are available both on the web pages of phytozome (v10) and at the PlantGDB web resource. The genomic and EST sequences of *Arabidopsis* came from the *Arabidopsis* Genome Initiative (AGI) [31]. The data are available both on the web pages of The *Arabidopsis* Information Resource (TAIR) (http://www.arabidopsis.org/) and at the PlantGDB web resource.

### Genomic mapping of expressed sequence clusters

The ESTs of the three species *Volvox*, *Chlamydomonas* and *Arabidopsis* were aligned to the corresponding genomic contigs and genome sequences using BLAT, a BLAST-like alignment tool [67]. To avoid double counting of paralogs, we only used the best alignment. Sequences with less than 95% identity were removed from further analysis. The resulting alignments were then clustered by genomic location where each cluster is formed by the set of all ESTs which overlap at a given location. The splice site consensus sequences (GT/AG) were identified and sequences without splice site consensus sequence were also removed. Subsequently, an alternative splicing graph of potential splice variants was constructed. An intron in the splice graph was constructed when there was EST evidence of a transcript with appropriate splice sites.

### Alternative splicing analysis

From the splice graphs, we identified alternatively spliced isoforms. Isoforms with alternative transcription starts or ends were not considered further in this paper. The isoforms were divided into four major groups of alternative splicing events: exon skipping, intron retention, alternative 5′ and alternative 3′ splicing. This was obtained by searching for patterns of exons and introns that are consistent with these events. In addition, there were instances of more complex splice forms, which were not covered by the above four simple alternative splicing events. To obtain high quality events, only events were included where the amount of sequence evidence for both splice alternatives was within ten times of each other. This was to exclude spurious events due to a small number of ESTs.

### Primer design

For verification of alternative splicing events, primers were designed such that they have similar melting temperatures, allowing both primers to anneal roughly at the same temperature in RT-PCR and real-time RT-PCR experiments. For each candidate gene, three pairs of primers were chosen to amplify the first and the second splice variant separately and also both splice variants together in one reaction tube (Figure 4 and Additional file 1: Table S3). The primers were designed using the primer analysis software Oligo 6 (Molecular Biology Insights, Cascade, CO), DNASIS (version 7.00; Hitachi Software Engineering, South San Francisco, CA), or Primer Express (Applied Biosystems, Foster City, CA).

### Isolation of total RNA

Total RNA was extracted from 1 g frozen algae using 10 ml of the phenol-based TRI Reagent (Sigma-Aldrich, St. Louis, MO) and 3 ml trichloromethane. RNA was precipitated from the aqueous phase by addition of isopropanol. All preparation and handling steps of RNA took place under RNase-free conditions. After final washing steps with ethanol, RNA was dissolved in RNase-free water and stored at -70°C until used. RNA quantity and purity were determined by measuring the 260/280 ratio using an UV/visible spectrophotometer (Ultrospec 2100 pro, GE Healthcare, Uppsala, Sweden). The quality of RNA was verified by electrophoresis of 0.5 μg RNA in a 1.3% agarose-formaldehyde gel.

### RT-PCR

1 μg of extracted total RNA was treated with 5 units of DNase I (Promega, Madison, WI) for 15 min at room temperature and then DNase I was inactivated at 75°C for 10 min. 1 unit of M-MLV Reverse Transcriptase (Promega, Madison, WI) and 1 μM of reverse primer were added for synthesis of the first strand cDNA at 50°C for 60 min (Figure 4, Additional file 1: Table S1). The synthesized cDNA was used as a template for PCR according to standard protocols [161].

### Quantitative real-time RT-PCR

Quantitative real-time RT-PCR was performed on a DNA Engine Opticon Continuous Fluorescence Detection System (MJ Research) using the SensiMix one-step kit (Quantace) as described previously [78]. 300 ng total RNA was used per 25 μl of reaction volume. The thermal cycling conditions were identical for all quantification experiments, i.e. after the first cDNA synthesis at 50°C for 30 min, DNA polymerase was activated at 95°C for 10 min, followed by 40 cycles of denaturation at 95°C for 20 s, annealing at 55°C for 30 s and extension at 72°C for 40 s. The expression levels of splice variants were calculated in relation to a reference gene, the housekeeping gene *actin* [76, 78, 162], using the 2^-ΔΔCt^ method [163, 164]. The Ct, ∆Ct, and ∆∆Ct values were calculated as described previously [78]. All real-time RT-PCR experiments were carried out in triplicate together with controls lacking RT or template. The final products of all real-time RT-PCR reactions were visualized using agarose gel electrophoresis to ensure amplification of a single product of the correct size.

## Electronic supplementary material

Additional file 1: **Supplemental Material.** Supplemental Figures S1 to S12 Supplemental Tables S1 to S4 and Supplemental References. (PDF 4 MB)

## Abbreviations

EST: Expressed sequence tags
UTR: Untranslated region
mRNA: Messenger mRNA
RT-PCR: Reverse transcription polymerase chain reaction.

## References

[1] Chen L, Bush SJ, Tovar-Corona JM, Castillo-Morales A, Urrutia AO (2014). Correcting for differential transcript coverage reveals a strong relationship between alternative splicing and organism complexity. Mol Biol Evol.

[2] Stamm S, Ben-Ari S, Rafalska I, Tang Y, Zhang Z, Toiber D, Thanaraj TA, Soreq H (2005). Function of alternative splicing. Gene.

[3] Keren H, Lev-Maor G, Ast G (2010). Alternative splicing and evolution: diversification, exon definition and function. Nat Rev Genet.

[4] Reddy AS (2001). Nuclear pre-mRNA splicing in plants. Crit Rev Plant Sci.

[5] Reddy AS (2007). Alternative splicing of pre-messenger RNAs in plants in the genomic era. Annu Rev Plant Biol.

[6] Gassmann W (2008). Alternative splicing in plant defense. Curr Top Microbiol Immunol.

[7] Cáceres JF, Kornblihtt AR (2002). Alternative splicing: multiple control mechanisms and involvement in human disease. Trends Genet.

[8] Grabowski PJ (1998). Splicing regulation in neurons: tinkering with cell-specific control. Cell.

[9] Grabowski PJ, Black DL (2001). Alternative RNA splicing in the nervous system. Prog Neurobiol.

[10] Stamm S, Zhang MQ, Marr TG, Helfman DM (1994). A sequence compilation and comparison of exons that are alternatively spliced in neurons. Nucleic Acids Res.

[11] Licatalosi DD, Darnell RB (2010). RNA processing and its regulation: global insights into biological networks. Nat Rev Genet.

[12] Black DL (2003). Mechanisms of alternative pre-messenger RNA splicing. Annu Rev Biochem.

[13] Johnson JM, Castle J, Garrett-Engele P, Kan Z, Loerch PM, Armour CD, Santos R, Schadt EE, Stoughton R, Shoemaker DD (2003). Genome-wide survey of human alternative pre-mRNA splicing with exon junction microarrays. Science.

[14] Kampa D, Cheng J, Kapranov P, Yamanaka M, Brubaker S, Cawley S, Drenkow J, Piccolboni A, Bekiranov S, Helt G, Tammana H, Gingeras TR (2004). Novel RNAs identified from an in-depth analysis of the transcriptome of human chromosomes 21 and 22. Genome Res.

[15] Pan Q, Shai O, Lee LJ, Frey BJ, Blencowe BJ (2008). Deep surveying of alternative splicing complexity in the human transcriptome by high-throughput sequencing. Nat Genet.

[16] Wang ET, Sandberg R, Luo S, Khrebtukova I, Zhang L, Mayr C, Kingsmore SF, Schroth GP, Burge CB (2008). Alternative isoform regulation in human tissue transcriptomes. Nature.

[17] Graveley BR, Brooks AN, Carlson JW, Duff MO, Landolin JM, Yang L, Artieri CG, van Baren MJ, Boley N, Booth BW, Brown JB, Cherbas L, Davis CA, Dobin A, Li R, Lin W, Malone JH, Mattiuzzo NR, Miller D, Sturgill D, Tuch BB, Zaleski C, Zhang D, Blanchette M, Dudoit S, Eads B, Green RE, Hammonds A, Jiang L, Kapranov P (2011). The developmental transcriptome of *Drosophila melanogaster*. Nature.

[18] Filichkin SA, Priest HD, Givan SA, Shen R, Bryant DW, Fox SE, Wong WK, Mockler TC (2010). Genome-wide mapping of alternative splicing in *Arabidopsis thaliana*. Genome Res.

[19] Marquez Y, Brown JW, Simpson C, Barta A, Kalyna M (2012). Transcriptome survey reveals increased complexity of the alternative splicing landscape in *Arabidopsis*. Genome Res.

[20] Barash Y, Calarco JA, Gao W, Pan Q, Wang X, Shai O, Blencowe BJ, Frey BJ (2010). Deciphering the splicing code. Nature.

[21] Ramani AK, Calarco JA, Pan Q, Mavandadi S, Wang Y, Nelson AC, Lee LJ, Morris Q, Blencowe BJ, Zhen M, Fraser AG (2011). Genome-wide analysis of alternative splicing in *Caenorhabditis elegans*. Genome Res.

[22] Zhu W, Schlueter SD, Brendel V (2003). Refined annotation of the *Arabidopsis* genome by complete expressed sequence tag mapping. Plant Physiol.

[23] Iida K, Seki M, Sakurai T, Satou M, Akiyama K, Toyoda T, Konagaya A, Shinozaki K (2004). Genome-wide analysis of alternative pre-mRNA splicing in *Arabidopsis thaliana* based on full-length cDNA sequences. Nucleic Acids Res.

[24] Campbell MA, Haas BJ, Hamilton JP, Mount SM, Buell CR (2006). Comprehensive analysis of alternative splicing in rice and comparative analyses with *Arabidopsis*. BMC Genomics.

[25] Hori K, Watanabe Y (2007). Context analysis of termination codons in mRNA that are recognized by plant NMD. Plant Cell Physiol.

[26] Barbazuk WB, Fu Y, McGinnis KM (2008). Genome-wide analyses of alternative splicing in plants: opportunities and challenges. Genome Res.

[27] Goodall GJ, Filipowicz W (1991). Different effects of intron nucleotide composition and secondary structure on pre-mRNA splicing in monocot and dicot plants. EMBO J.

[28] McCullough AJ, Schuler MA (1993). AU-rich intronic elements affect pre-mRNA 5′ splice site selection in *Drosophila melanogaster*. Mol Cell Biol.

[29] Lim LP, Burge CB (2001). A computational analysis of sequence features involved in recognition of short introns. Proc Natl Acad Sci U S A.

[30] Goodall GJ, Filipowicz W (1989). The AU-rich sequences present in the introns of plant nuclear pre-mRNAs are required for splicing. Cell.

[31] Arabidopsis Genome Initiative (2000). Analysis of the genome sequence of the lowering plant *Arabidopsis thaliana*. Nature.

[32] Lander ES, Linton LM, Birren B, Nusbaum C, Zody MC, Baldwin J, Devon K, Dewar K, Doyle M, FitzHugh W, Funke R, Gage D, Harris K, Heaford A, Howland J, Kann L, Lehoczky J, LeVine R, McEwan P, McKernan K, Meldrim J, Mesirov JP, Miranda C, Morris W, Naylor J, Raymond C, Rosetti M, Santos R, Sheridan A, Sougnez C (2001). Initial sequencing and analysis of the human genome. Nature.

[33] Alexandrov NN, Troukhan ME, Brover VV, Tatarinova T, Flavell RB, Feldmann KA (2006). Features of *Arabidopsis* genes and genome discovered using full-length cDNAs. Plant Mol Biol.

[34] Wang BB, Brendel V (2006). Genomewide comparative analysis of alternative splicing in plants. Proc Natl Acad Sci U S A.

[35] White O, Soderlund C, Shanmugan P, Fields C (1992). Information contents and dinucleotide compositions of plant intron sequences vary with evolutionary origin. Plant Mol Biol.

[36] Yu J, Hu S, Wang J, Wong GK, Li S, Liu B, Deng Y, Dai L, Zhou Y, Zhang X, Cao M, Liu J, Sun J, Tang J, Chen Y, Huang X, Lin W, Ye C, Tong W, Cong L, Geng J, Han Y, Li L, Li W, Hu G, Li J, Liu Z, Qi Q, Li T, Wang X (2002). A draft sequence of the rice genome (*Oryza sativa* L. ssp. *indica*). Science.

[37] Baek JM, Han P, Iandolino A, Cook DR (2008). Characterization and comparison of intron structure and alternative splicing between *Medicago truncatula*, *Populus trichocarpa,* Arabidopsis and rice. Plant Mol Biol.

[38] Luehrsen KR, Taha S, Walbot V (1994). Nuclear pre-mRNA processing in higher plants. Prog Nucleic Acid Res Mol Biol.

[39] Jordan T, Schornack S, Lahaye T (2002). Alternative splicing of transcripts encoding Toll-like plant resistance proteins - what’s the functional relevance to innate immunity?. Trends Plant Sci.

[40] Tanabe N, Yoshimura K, Kimura A, Yabuta Y, Shigeoka S (2007). Differential expression of alternatively spliced mRNAs of *Arabidopsis* SR protein homologs, atSR30 and atSR45a, in response to environmental stress. Plant Cell Physiol.

[41] Egawa C, Kobayashi F, Ishibashi M, Nakamura T, Nakamura C, Takumi S (2006). Differential regulation of transcript accumulation and alternative splicing of a DREB2 homolog under abiotic stress conditions in common wheat. Genes Genet Syst.

[42] Christensen AH, Sharrock RA, Quail PH (1992). Maize polyubiquitin genes: structure, thermal perturbation of expression and transcript splicing, and promoter activity following transfer to protoplasts by electroporation. Plant Mol Biol.

[43] Hopf N, Plesofsky-Vig N, Brambl R (1992). The heat shock response of pollen and other tissues of maize. Plant Mol Biol.

[44] Mazzucotelli E, Mastrangelo AA, Crosatti C, Guerra D, Stanca AM, Cattivelli L (2008). Abiotic stress response in plants: when post-transcriptional and post-translational regulations control transcription. Plant Sci.

[45] Ali GS, Reddy AS (2008). Regulation of alternative splicing of pre-mRNAs by stresses. Curr Top Microbiol Immunol.

[46] Syed NH, Kalyna M, Marquez Y, Barta A, Brown JW (2012). Alternative splicing in plants - coming of age. Trends Plant Sci.

[47] James AB, Syed NH, Bordage S, Marshall J, Nimmo GA, Jenkins GI, Herzyk P, Brown JW, Nimmo HG (2012). Alternative splicing mediates responses of the Arabidopsis circadian clock to temperature changes. Plant Cell.

[48] Huber O, Sumper M (1994). Algal-CAMs: isoforms of a cell adhesion molecule in embryos of the alga *Volvox* with homology to *Drosophila* fasciclin I. EMBO J.

[49] Kianianmomeni A, Nematollahi G, Hallmann A (2008). A gender-specific retinoblastoma-related protein in *Volvox carteri* implies a role for the retinoblastoma protein family in sexual development. Plant Cell.

[50] Ferris P, Olson BJ, De Hoff PL, Douglass S, Casero D, Prochnik S, Geng S, Rai R, Grimwood J, Schmutz J, Nishii I, Hamaji T, Nozaki H, Pellegrini M, Umen JG (2010). Evolution of an expanded sex-determining locus in *Volvox*. Science.

[51] Fuhrmann M, Stahlberg A, Govorunova E, Rank S, Hegemann P (2001). The abundant retinal protein of the *Chlamydomonas* eye is not the photoreceptor for phototaxis and photophobic responses. J Cell Sci.

[52] Schroda M, Vallon O, Whitelegge JP, Beck CF, Wollman FA (2001). The chloroplastic GrpE homolog of *Chlamydomonas*: two isoforms generated by differential splicing. Plant Cell.

[53] Labadorf A, Link A, Rogers MF, Thomas J, Reddy AS, Ben-Hur A (2010). Genome-wide analysis of alternative splicing in *Chlamydomonas reinhardtii*. BMC Genomics.

[54] Herron MD, Hackett JD, Aylward FO, Michod RE (2009). Triassic origin and early radiation of multicellular volvocine algae. Proc Natl Acad Sci U S A.

[55] Sanderson MJ (2003). Molecular data from 27 proteins do not support a Precambrian origin of land plants. Am J Bot.

[56] Peterson KJ, Butterfield NJ (2005). Origin of the Eumetazoa: testing ecological predictions of molecular clocks against the Proterozoic fossil record. Proc Natl Acad Sci U S A.

[57] Kirk DL (2005). A twelve-step program for evolving multicellularity and a division of labor. Bioessays.

[58] Prochnik SE, Umen J, Nedelcu AM, Hallmann A, Miller SM, Nishii I, Ferris P, Kuo A, Mitros T, Fritz-Laylin LK, Hellsten U, Chapman J, Simakov O, Rensing SA, Terry A, Pangilinan J, Kapitonov V, Jurka J, Salamov A, Shapiro H, Schmutz J, Grimwood J, Lindquist E, Lucas S, Grigoriev IV, Schmitt R, Kirk D, Rokhsar DS (2010). Genomic analysis of organismal complexity in the multicellular green alga *Volvox carteri*. Science.

[59] Merchant SS, Prochnik SE, Vallon O, Harris EH, Karpowicz SJ, Witman GB, Terry A, Salamov A, Fritz-Laylin LK, Marechal-Drouard L, Marshall WF, Qu LH, Nelson DR, Sanderfoot AA, Spalding MH, Kapitonov VV, Ren Q, Ferris P, Lindquist E, Shapiro H, Lucas SM, Grimwood J, Schmutz J, Cardol P, Cerutti H, Chanfreau G, Chen CL, Cognat V, Croft MT, Dent R (2007). The *Chlamydomonas* genome reveals the evolution of key animal and plant functions. Science.

[60] Pennisi E (2010). *Volvox* genome shows it doesn’t take much to be multicellular. Science.

[61] Irimia M, Penny D, Roy SW (2007). Coevolution of genomic intron number and splice sites. Trends Genet.

[62] Holland LZ, Short S (2010). Alternative splicing in development and function of chordate endocrine systems: a focus on Pax genes. Integr Comp Biol.

[63] Grabowski P (2011). Alternative splicing takes shape during neuronal development. Curr Opin Genet Dev.

[64] Lozada-Chávez I, Stadler PF, Prohaska SJ (2011). “Hypothesis for the modern RNA world”: a pervasive non-coding RNA-based genetic regulation is a prerequisite for the emergence of multicellular complexity. Orig Life Evol Biosph.

[65] Kim E, Magen A, Ast G (2007). Different levels of alternative splicing among eukaryotes. Nucleic Acids Res.

[66] Roy M, Kim N, Xing Y, Lee C (2008). The effect of intron length on exon creation ratios during the evolution of mammalian genomes. RNA.

[67] Kent WJ (2002). BLAT - the BLAST-like alignment tool. Genome Res.

[68] Sparks ME, Brendel V (2005). Incorporation of splice site probability models for non-canonical introns improves gene structure prediction in plants. Bioinformatics.

[69] Yu AY, Houry WA (2007). ClpP: a distinctive family of cylindrical energy-dependent serine proteases. FEBS Lett.

[70] Majeran W, Friso G, van Wijk KJ, Vallon O (2005). The chloroplast ClpP complex in *Chlamydomonas reinhardtii* contains an unusual high molecular mass subunit with a large apical domain. FEBS J.

[71] Ekici OD, Paetzel M, Dalbey RE (2008). Unconventional serine proteases: variations on the catalytic Ser/His/Asp triad configuration. Protein Sci.

[72] Wang J, Hartling JA, Flanagan JM (1997). The structure of ClpP at 2.3 Å resolution suggests a model for ATP-dependent proteolysis. Cell.

[73] Bove J, Kim CY, Gibson CA, Assmann SM (2008). Characterization of wound-responsive RNA-binding proteins and their splice variants in *Arabidopsis*. Plant Mol Biol.

[74] Moll AG, Lindenmeyer MT, Kretzler M, Nelson PJ, Zimmer R, Cohen CD (2009). Transcript-specific expression profiles derived from sequence-based analysis of standard microarrays. PLoS One.

[75] Chamberlain KL, Miller SH, Keller LR (2008). Gene expression profiling of flagellar disassembly in *Chlamydomonas reinhardtii*. Genetics.

[76] Amon P, Haas E, Sumper M (1998). The sex-inducing pheromone and wounding trigger the same set of genes in the multicellular green alga *Volvox*. Plant Cell.

[77] Hallmann A (2006). The pherophorins: common, versatile building blocks in the evolution of extracellular matrix architecture in Volvocales. Plant J.

[78] Nematollahi G, Kianianmomeni A, Hallmann A (2006). Quantitative analysis of cell-type specific gene expression in the green alga *Volvox carteri*. BMC Genomics.

[79] Fraga D, Meulia T, Fenster S, Gallagher SR, Wiley EA (2008). Real-Time PCR. Current Protocols Essential Laboratory Techniques.

[80] Hilgenfeld R (1995). Regulatory GTPases. Curr Opin Struct Biol.

[81] Agirrezabala X, Frank J (2009). Elongation in translation as a dynamic interaction among the ribosome, tRNA, and elongation factors EF-G and EF-Tu. Q Rev Biophys.

[82] Kjeldgaard M, Nyborg J (1992). Refined structure of elongation factor EF-Tu from *Escherichia coli*. J Mol Biol.

[83] Kawashima T, Berthet-Colominas C, Wulff M, Cusack S, Leberman R (1996). The structure of the *Escherichia coli* EF-Tu.EF-Ts complex at 2.5 Å resolution. Nature.

[84] Bourne HR, Sanders DA, McCormick F (1991). The GTPase superfamily: conserved structure and molecular mechanism. Nature.

[85] Berchtold H, Reshetnikova L, Reiser CO, Schirmer NK, Sprinzl M, Hilgenfeld R (1993). Crystal structure of active elongation factor Tu reveals major domain rearrangements. Nature.

[86] Nissen P, Kjeldgaard M, Thirup S, Polekhina G, Reshetnikova L, Clark BF, Nyborg J (1995). Crystal structure of the ternary complex of Phe-tRNAPhe, EF-Tu, and a GTP analog. Science.

[87] Adams MW (1990). The structure and mechanism of iron-hydrogenases. Biochim Biophys Acta.

[88] Przybyla AE, Robbins J, Menon N, Peck HD (1992). Structure-function relationships among the nickel-containing hydrogenases. FEMS Microbiol Rev.

[89] Happe T, Kaminski A (2002). Differential regulation of the Fe-hydrogenase during anaerobic adaptation in the green alga *Chlamydomonas reinhardtii*. Eur J Biochem.

[90] Forestier M, King P, Zhang L, Posewitz M, Schwarzer S, Happe T, Ghirardi ML, Seibert M (2003). Expression of two [Fe]-hydrogenases in *Chlamydomonas reinhardtii* under anaerobic conditions. Eur J Biochem.

[91] Peters JW, Lanzilotta WN, Lemon BJ, Seefeldt LC (1998). X-ray crystal structure of the Fe-only hydrogenase (CpI) from *Clostridium pasteurianum* to 1.8 Angstrom resolution. Science.

[92] Shimizu T, Inoue T, Shiraishi H (2002). Cloning and characterization of novel extensin-like cDNAs that are expressed during late somatic cell phase in the green alga *Volvox carteri*. Gene.

[93] Kinoshita T, Fukuzawa H, Shimada T, Saito T, Matsuda Y (1992). Primary structure and expression of a gamete lytic enzyme in *Chlamydomonas reinhardtii*: similarity of functional domains to matrix metalloproteases. Proc Natl Acad Sci U S A.

[94] Hallmann A, Amon P, Godl K, Heitzer M, Sumper M (2001). Transcriptional activation by the sexual pheromone and wounding: a new gene family from *Volvox* encoding modular proteins with (hydroxy)proline-rich and metalloproteinase homology domains. Plant J.

[95] Heitzer M, Hallmann A (2002). An extracellular matrix-localized metalloproteinase with an exceptional QEXXH metal binding site prefers copper for catalytic activity. J Biol Chem.

[96] Hooper NM (1994). Families of zinc metalloproteases. FEBS Lett.

[97] Soejima H, Zhao W, Mukai T (2005). Epigenetic silencing of the MGMT gene in cancer. Biochem Cell Biol.

[98] Kaina B, Christmann M, Naumann S, Roos WP (2007). MGMT: key node in the battle against genotoxicity, carcinogenicity and apoptosis induced by alkylating agents. DNA Repair (Amst).

[99] Margison GP, Povey AC, Kaina B, Santibáñez Koref MF (2003). Variability and regulation of O^6^-alkylguanine-DNA alkyltransferase. Carcinogenesis.

[100] Daniels DS, Mol CD, Arvai AS, Kanugula S, Pegg AE, Tainer JA (2000). Active and alkylated human AGT structures: a novel zinc site, inhibitor and extrahelical base binding. EMBO J.

[101] Daniels DS, Woo TT, Luu KX, Noll DM, Clarke ND, Pegg AE, Tainer JA (2004). DNA binding and nucleotide flipping by the human DNA repair protein AGT. Nat Struct Mol Biol.

[102] Duguid EM, Rice PA, He C (2005). The structure of the human AGT protein bound to DNA and its implications for damage detection. J Mol Biol.

[103] Dreyfuss G, Kim VN, Kataoka N (2002). Messenger-RNA-binding proteins and the messages they carry. Nat Rev Mol Cell Biol.

[104] Maris C, Dominguez C, Allain FH (2005). The RNA recognition motif, a plastic RNA-binding platform to regulate post-transcriptional gene expression. FEBS J.

[105] Cléry A, Blatter M, Allain FH (2008). RNA recognition motifs: boring? not quite. Curr Opin Struct Biol.

[106] Oubridge C, Ito N, Evans PR, Teo CH, Nagai K (1994). Crystal structure at 1.92 Å resolution of the RNA-binding domain of the U1A spliceosomal protein complexed with an RNA hairpin. Nature.

[107] Low SC, Berry MJ (1996). Knowing when not to stop: selenocysteine incorporation in eukaryotes. Trends Biochem Sci.

[108] Wang P, Heitman J (2005). The cyclophilins. Genome Biol.

[109] Maleszka R, Lupas A, Hanes SD, Miklos GL (1997). The *dodo* gene family encodes a novel protein involved in signal transduction and protein folding. Gene.

[110] Horowitz DS, Lee EJ, Mabon SA, Misteli T (2002). A cyclophilin functions in pre-mRNA splicing. EMBO J.

[111] Ingelfinger D, Gothel SF, Marahiel MA, Reidt U, Ficner R, Luhrmann R, Achsel T (2003). Two protein-protein interaction sites on the spliceosome-associated human cyclophilin CypH. Nucleic Acids Res.

[112] He Z, Li L, Luan S (2004). Immunophilins and parvulins. superfamily of peptidyl prolyl isomerases in Arabidopsis. Plant Physiol.

[113] Romano P, Gray J, Horton P, Luan S (2005). Plant immunophilins: functional versatility beyond protein maturation. New Phytol.

[114] Kallen J, Spitzfaden C, Zurini MG, Wider G, Widmer H, Wuthrich K, Walkinshaw MD (1991). Structure of human cyclophilin and its binding site for cyclosporin A determined by X-ray crystallography and NMR spectroscopy. Nature.

[115] Pflügl G, Kallen J, Schirmer T, Jansonius JN, Zurini MG, Walkinshaw MD (1993). X-ray structure of a decameric cyclophilin-cyclosporin crystal complex. Nature.

[116] Zydowsky LD, Etzkorn FA, Chang HY, Ferguson SB, Stolz LA, Ho SI, Walsh CT (1992). Active site mutants of human cyclophilin A separate peptidyl-prolyl isomerase activity from cyclosporin A binding and calcineurin inhibition. Protein Sci.

[117] Cardenas ME, Lim E, Heitman J (1995). Mutations that perturb cyclophilin A ligand binding pocket confer cyclosporin A resistance in *Saccharomyces cerevisiae*. J Biol Chem.

[118] Tam LW, Kirk DL (1991). Identification of cell-type-specific genes of *Volvox carteri* and characterization of their expression during the asexual life cycle. Dev Biol.

[119] Meissner M, Stark K, Cresnar B, Kirk DL, Schmitt R (1999). *Volvox* germline-specific genes that are putative targets of RegA repression encode chloroplast proteins. Curr Genet.

[120] Mayfield SP, Bennoun P, Rochaix JD (1987). Expression of the nuclear encoded OEE1 protein is required for oxygen evolution and stability of photosystem II particles in *Chlamydomonas reinhardtii*. EMBO J.

[121] Yi X, McChargue M, Laborde S, Frankel LK, Bricker TM (2005). The manganese-stabilizing protein is required for photosystem II assembly/stability and photoautotrophy in higher plants. J Biol Chem.

[122] García-Gimeno MA, Muñoz I, Ariño J, Sanz P (2003). Molecular characterization of Ypi1, a novel *Saccharomyces cerevisiae* type 1 protein phosphatase inhibitor. J Biol Chem.

[123] Pazour GJ, Agrin N, Leszyk J, Witman GB (2005). Proteomic analysis of a eukaryotic cilium. J Cell Biol.

[124] Modrek B, Resch A, Grasso C, Lee C (2001). Genome-wide detection of alternative splicing in expressed sequences of human genes. Nucleic Acids Res.

[125] Zavolan M, Kondo S, Schonbach C, Adachi J, Hume DA, Hayashizaki Y, Gaasterland T (2003). Impact of alternative initiation, splicing, and termination on the diversity of the mRNA transcripts encoded by the mouse transcriptome. Genome Res.

[126] Gupta S, Zink D, Korn B, Vingron M, Haas SA (2004). Genome wide identification and classification of alternative splicing based on EST data. Bioinformatics.

[127] Okazaki Y, Furuno M, Kasukawa T, Adachi J, Bono H, Kondo S, Nikaido I, Osato N, Saito R, Suzuki H, Yamanaka I, Kiyosawa H, Yagi K, Tomaru Y, Hasegawa Y, Nogami A, Schönbach C, Gojobori T, Baldarelli R, Hill DP, Bult C, Hume DA, Quackenbush J, Schriml LM, Kanapin A, Matsuda H, Batalov S, Beisel KW, Blake JA, Bradt D (2002). Analysis of the mouse transcriptome based on functional annotation of 60,770 full-length cDNAs. Nature.

[128] Nilsen TW, Graveley BR (2010). Expansion of the eukaryotic proteome by alternative splicing. Nature.

[129] Klaff P, Riesner D, Steger G (1996). RNA structure and the regulation of gene expression. Plant Mol Biol.

[130] Nackley AG, Shabalina SA, Tchivileva IE, Satterfield K, Korchynskyi O, Makarov SS, Maixner W, Diatchenko L (2006). Human catechol-O-methyltransferase haplotypes modulate protein expression by altering mRNA secondary structure. Science.

[131] Kudla G, Murray AW, Tollervey D, Plotkin JB (2009). Coding-sequence determinants of gene expression in *Escherichia coli*. Science.

[132] Gommans WM, Mullen SP, Maas S (2009). RNA editing: a driving force for adaptive evolution?. Bioessays.

[133] Surdej P, Riedl A, Jacobs-Lorena M (1994). Regulation of mRNA stability in development. Annu Rev Genet.

[134] Gamazon ER, Stranger BE (2014). Genomics of alternative splicing: evolution, development and pathophysiology. Hum Genet.

[135] Fedorova L, Fedorov A (2005). Puzzles of the human genome: why do we need our introns?. Curr Genomics.

[136] Galante PA, Sakabe NJ, Kirschbaum-Slager N, de Souza SJ (2004). Detection and evaluation of intron retention events in the human transcriptome. RNA.

[137] Stamm S, Zhu J, Nakai K, Stoilov P, Stoss O, Zhang MQ (2000). An alternative-exon database and its statistical analysis. DNA Cell Biol.

[138] Zheng CL, Fu XD, Gribskov M (2005). Characteristics and regulatory elements defining constitutive splicing and different modes of alternative splicing in human and mouse. RNA.

[139] McGuire AM, Pearson MD, Neafsey DE, Galagan JE (2008). Cross-kingdom patterns of alternative splicing and splice recognition. Genome Biol.

[140] Kandul NP, Noor MA (2009). Large introns in relation to alternative splicing and gene evolution: a case study of *Drosophila* bruno-3. BMC Genet.

[141] Fox-Walsh KL, Dou Y, Lam BJ, Hung SP, Baldi PF, Hertel KJ (2005). The architecture of pre-mRNAs affects mechanisms of splice-site pairing. Proc Natl Acad Sci U S A.

[142] Yanofsky MF, Ma H, Bowman JL, Drews GN, Feldmann KA, Meyerowitz EM (1990). The protein encoded by the *Arabidopsis* homeotic gene agamous resembles transcription factors. Nature.

[143] Hong RL, Hamaguchi L, Busch MA, Weigel D (2003). Regulatory elements of the floral homeotic gene AGAMOUS identified by phylogenetic footprinting and shadowing. Plant Cell.

[144] Bruggmann R, Bharti AK, Gundlach H, Lai J, Young S, Pontaroli AC, Wei F, Haberer G, Fuks G, Du C, Raymond C, Estep MC, Liu R, Bennetzen JL, Chan AP, Rabinowicz PD, Quackenbush J, Barbazuk WB, Wing RA, Birren B, Nusbaum C, Rounsley S, Mayer KF, Messing J (2006). Uneven chromosome contraction and expansion in the maize genome. Genome Res.

[145] Kobayashi Y, Dokiya Y, Sugiura M, Niwa Y, Sugita M (2001). Genomic organization and organ-specific expression of a nuclear gene encoding phage-type RNA polymerase in *Nicotiana sylvestris*. Gene.

[146] Tadege M, Sheldon CC, Helliwell CA, Upadhyaya NM, Dennis ES, Peacock WJ (2003). Reciprocal control of flowering time by OsSOC1 in transgenic *Arabidopsis* and by FLC in transgenic rice. Plant Biotechnol J.

[147] Haddrill PR, Charlesworth B, Halligan DL, Andolfatto P (2005). Patterns of intron sequence evolution in *Drosophila* are dependent upon length and GC content. Genome Biol.

[148] Marais G, Nouvellet P, Keightley PD, Charlesworth B (2005). Intron size and exon evolution in *Drosophila*. Genetics.

[149] Irimia M, Rukov JL, Penny D, Roy SW (2007). Functional and evolutionary analysis of alternatively spliced genes is consistent with an early eukaryotic origin of alternative splicing. BMC Evol Biol.

[150] Luco RF, Allo M, Schor IE, Kornblihtt AR, Misteli T (2011). Epigenetics in alternative pre-mRNA splicing. Cell.

[151] Taneri B, Snyder B, Novoradovsky A, Gaasterland T (2004). Alternative splicing of mouse transcription factors affects their DNA-binding domain architecture and is tissue specific. Genome Biol.

[152] Umen JG, Goodenough UW (2001). Control of cell division by a retinoblastoma protein homolog in *Chlamydomonas*. Genes Dev.

[153] Umen JG (2011). Evolution of sex and mating loci: an expanded view from volvocine algae. Curr Opin Microbiol.

[154] Xiao YL, Smith SR, Ishmael N, Redman JC, Kumar N, Monaghan EL, Ayele M, Haas BJ, Wu HC, Town CD (2005). Analysis of the cDNAs of hypothetical genes on *Arabidopsis* chromosome 2 reveals numerous transcript variants. Plant Physiol.

[155] Melamud E, Moult J (2009). Stochastic noise in splicing machinery. Nucleic Acids Res.

[156] Pickrell JK, Pai AA, Gilad Y, Pritchard JK (2010). Noisy splicing drives mRNA isoform diversity in human cells. PLoS Genet.

[157] Resch A, Xing Y, Alekseyenko A, Modrek B, Lee C (2004). Evidence for a subpopulation of conserved alternative splicing events under selection pressure for protein reading frame preservation. Nucleic Acids Res.

[158] Adams CR, Stamer KA, Miller JK, McNally JG, Kirk MM, Kirk DL (1990). Patterns of organellar and nuclear inheritance among progeny of two geographically isolated strains of *Volvox carteri*. Curr Genet.

[159] Provasoli L, Pintner IJ, Tryon CA, Hartman RT (1959). Artificial media for fresh-water algae: problems and suggestions. The Ecology of Algae, a Symposium Held at the Pymatuning Laboratory of Field Biology on June 18 and 19, 1959.

[160] Starr RC, Jaenicke L (1974). Purification and characterization of the hormone initiating sexual morphogenesis in *Volvox carteri* f. *nagariensis* Iyengar. Proc Natl Acad Sci U S A.

[161] Sambrook J, Russell DW (2001). Molecular Cloning: A Laboratory Manual (3rd Edition), Vol. 1-3, 3rd edn.

[162] Cresnar B, Mages W, Müller K, Salbaum JM, Schmitt R (1990). Structure and expression of a single actin gene in *Volvox carteri*. Curr Genet.

[163] Bustin SA (2000). Absolute quantification of mRNA using real-time reverse transcription polymerase chain reaction assays. J Mol Endocrinol.

[164] Pfaffl MW (2001). A new mathematical model for relative quantification in real-time RT-PCR. Nucleic Acids Res.

