# Supplementary material for: Genome-wide analysis of alternative splicing in Volvox carteri
Source: BMC Genomics. 2014 Dec 16;15:1117. doi: 10.1186/1471-2164-15-1117 (PMC4378016; doi:10.1186/1471-2164-15-1117)
Supplement: Supplementary file 1 — Additional file 1: Supplemental Material. Supplemental Figures S1 to S12 Supplemental Tables S1 to S4 and Supplemental References. (PDF 4 MB) [file 12864_2014_6874_MOESM1_ESM.pdf]

Supplemental Material for
Genome-wide analysis of alternative splicing in *Volvox carteri*

Table of Contents

Supplemental Figures 2

Figure S1: Comparison protein isoforms encoded by *clpr2* 3

Figure S2: Comparison protein isoforms encoded by *efg8* 4

Figure S3: Comparison protein isoforms encoded by *hyd2* 5

Figure S4: Comparison protein isoforms encoded by *lgs2* 6

Figure S5: Comparison protein isoforms encoded by *mgmt* 7

Figure S6: Comparison protein isoforms encoded by *nrnp1* 8

Figure S7: Comparison protein isoforms encoded by *selEFf* 9

Figure S8: Comparison protein isoforms encoded by *cyn23* 10

Figure S9: Comparison protein isoforms encoded by *oee1* 11

Figure S10: Comparison protein isoforms encoded by *ppi1* 12

Figure S11: cDNA alignment of *cyn23* splice variants 13

Figure S12: Relationship between estimated alternative splicing (AS) rate and EST coverage in *Arabidopsis thaliana*, *Volvox carteri* and *Chlamydomonas reinhardtii* 14

Supplemental Tables 15

Table S1: Genome statistics of *Volvox* and other species 15

Table S2: List of samples genes for experimental verification 16

Table S3: Names as sequences of oligonucleotide primers 17

Table S4: Alternative splicing status of orthologous genes between *Volvox* and its close relative *Chlamydomonas* 18

Supplemental References 22

Supplemental Figures

The Supplemental Figures S1 to S10 show protein sequence alignments (part A) and schematic representations (part B) of alternative splice variants. The deduced amino acid sequences were aligned and coloured using GeneDoc 2.6 [3]. The upper sequence represents the amino acid sequence of the first alternative splice variant (V1) while the lower sequence represents the amino acid sequence of the second alternative splice variant (V2). Identical amino acids are shown by white letters on black background. The amino acids, for which similarity was conserved at more than 80%, are shown by white letters on grey background. The positions of the introns are marked by red arrowheads. The numbers indicate the amino acid position in (A). The scale bar indicates the length in amino acid residues in (B).

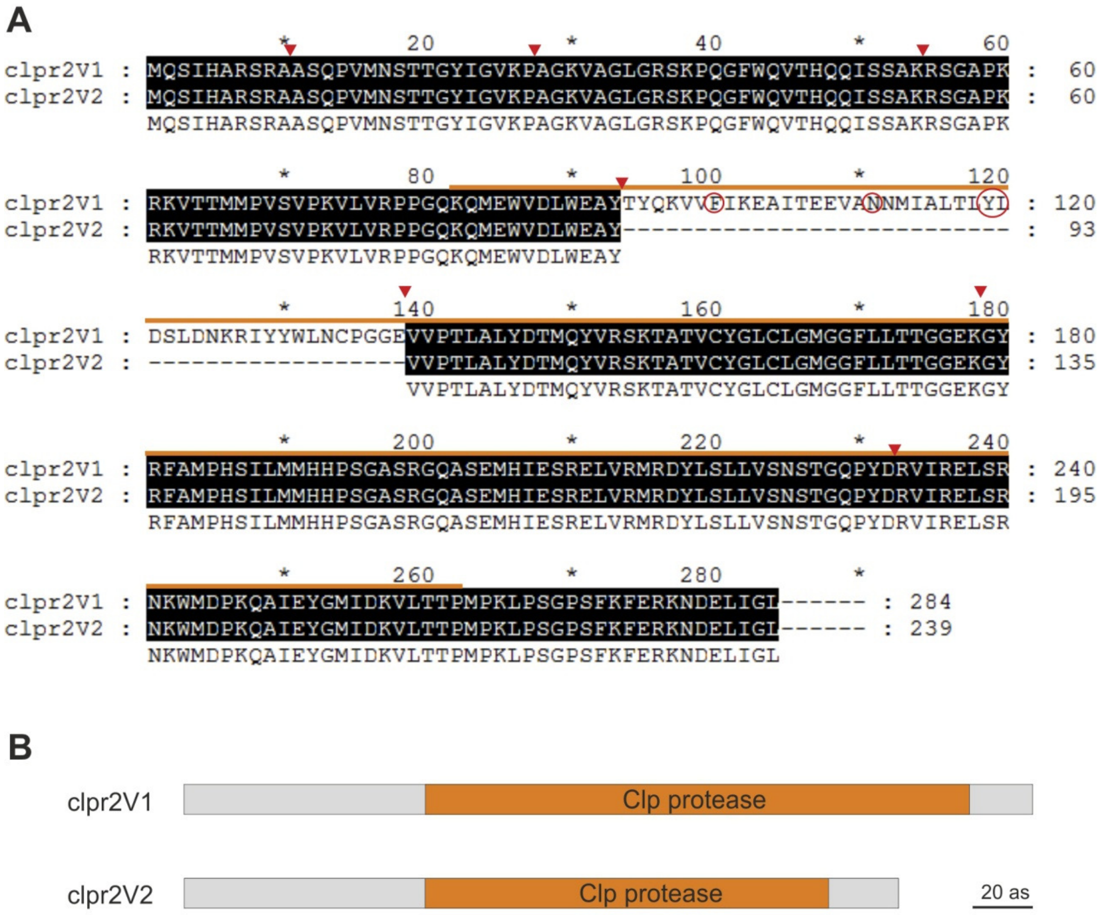

Figure S1: Comparison protein isoforms encoded by *clpr2*

Protein sequence alignment (A) and schematic representation (B) of the deduced amino acid sequences of two alternative splice variants (clpr2V1 and clpr2V2) of *clpr2*. The clp protease domain of clpr2V1, which is 181 amino acid residues in length, is shortened to 136 amino acid residues in clpr2V2. The conserved residues F100, N111, Y119 and L120, which are involved in α/β-type fold of the protein [6], are highlighted by the red circles. Thick orange line and orange box indicate clp protease domain in (A) and (B), respectively.

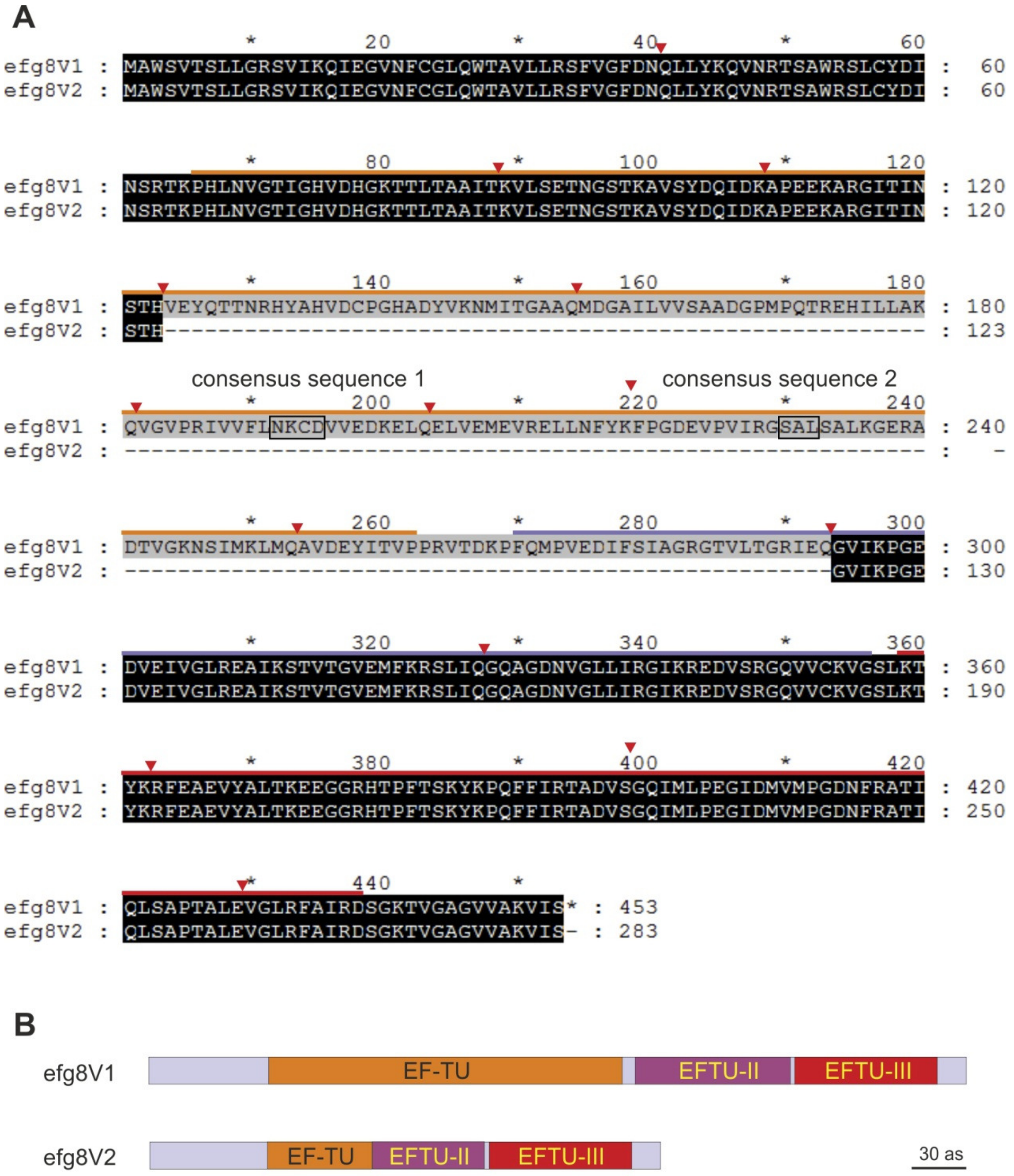

Figure S2: Comparison protein isoforms encoded by *efg8*

Protein sequence alignment (A) and schematic representation (B) of the deduced amino acid sequences of two alternative splice variants (efg8V1 and efg8V2) of *efg8*. efg8V1 possesses three elongation factor domains, namely EF-TU, EFTU-II and EFTU-III. EF-TU and EFTU-II domains are shortened in efg8V2. Furthermore, two consensus sequences [7], namely Asn-Lys-x-Asp (residues 192 to 195 of efg8V1) and Ser-Ala-Leu/Lys (residues 230 to 232 of efg8V1), which are important to bind guanine nucleotide [7], do not exist in efg8V2. EF-TU, EFTU-II and EFTU-III domains are highlighted in orange, purple and red in (A) and (B), in that order.

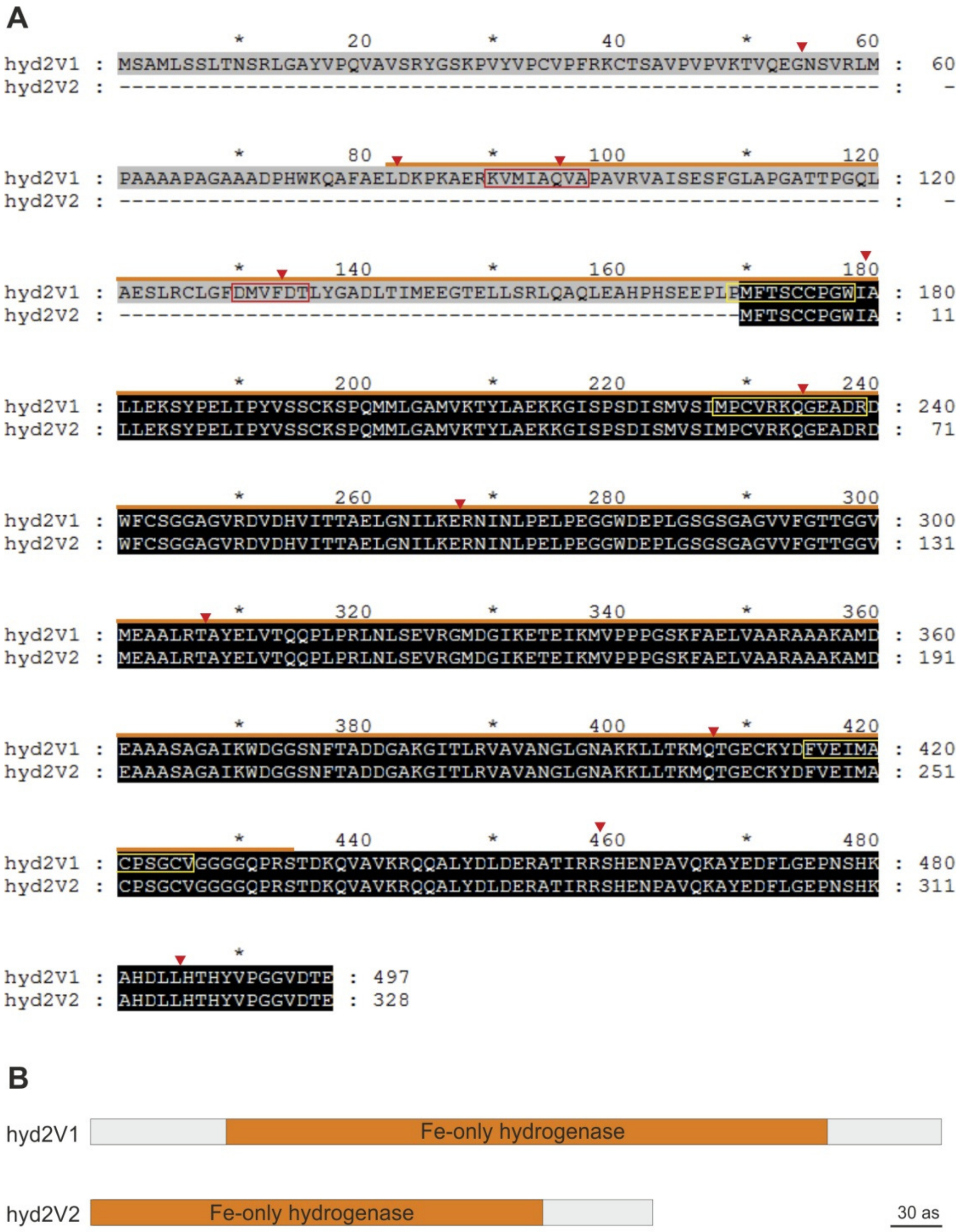

Figure S3: Comparison protein isoforms encoded by *hyd2*

Protein sequence alignment (A) and schematic representation (B) of the deduced amino acid sequences of two alternative splice variants (hyd2V1 and hyd2V2) of *hyd2*. The alternative splicing causes an N-terminal truncated Fe-only hydrogenase domain in hydV2. This second protein variant is 169 amino acid residues shorter than hyd2V1. The amino acid residues 90 to 97 and 130 to 135, which have been shown to form the β sheets around the active site [9], are highlighted by red boxes. Three motifs of the Fe-only hydrogenase active site, namely motif 1 (PMFTSCCPxW, amino acid residues 169 to 178), motif 2 (MPCxxKxxExxR, amino acid residues 228 to 239) and motif 3 (FxExMACxGxCV, amino acid residues 415 to 426) [10] are highlighted by yellow boxes. Thick orange line and orange box are highlighting the Fe-only hydrogenase domain in (A) and (B), respectively.

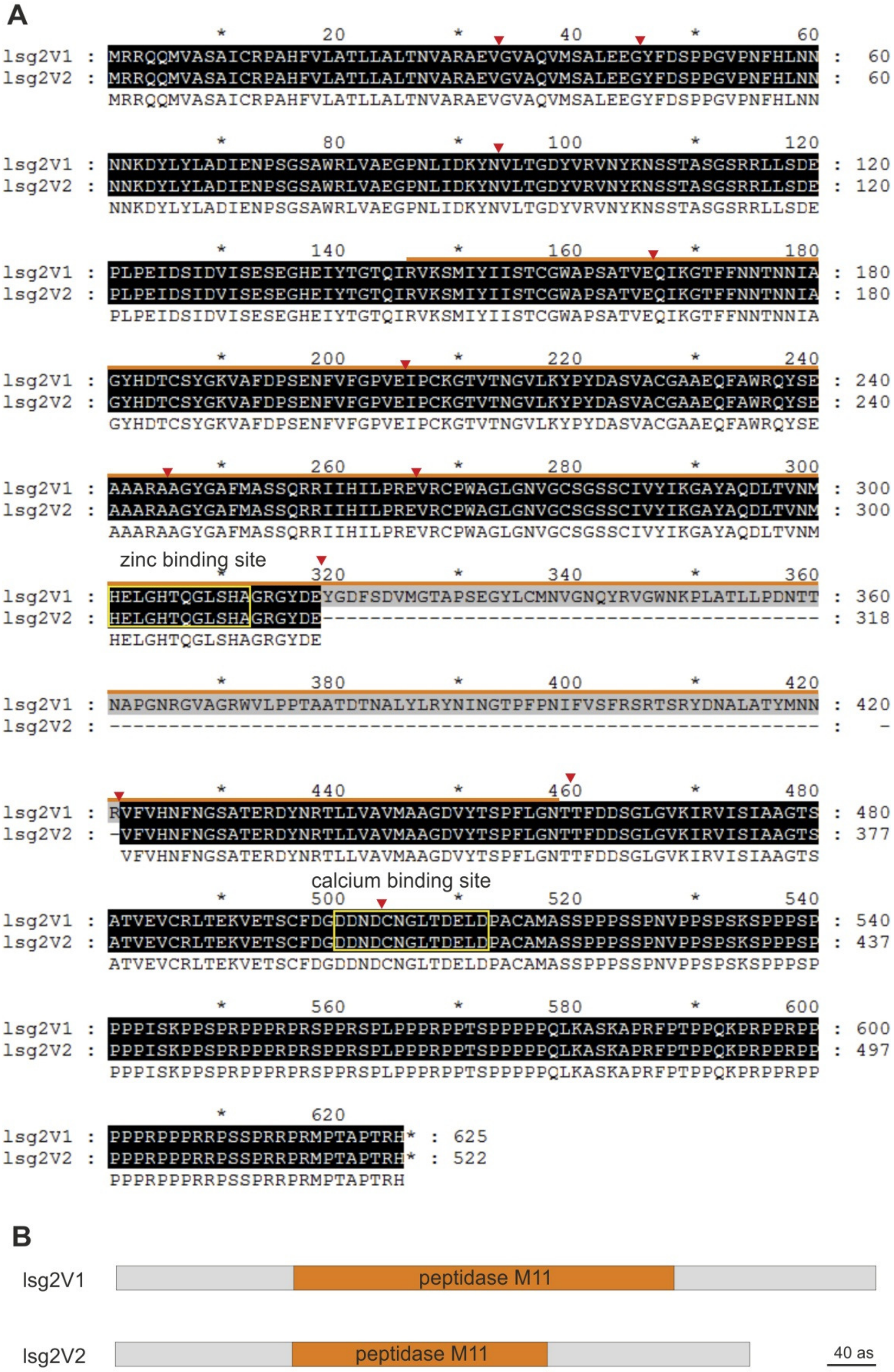

Figure S4: Comparison protein isoforms encoded by *lgs2*

Protein sequence alignment (A) and schematic representation (B) of the deduced amino acid sequences of two alternative splice variants (lsg2V1 and lsg2V2) of *lgs2*. Peptidase M11 domain of lsg2V2 is 103 amino acid residues shorter than of lsg2V1. Zinc and calcium binding sites of peptidase domain are highlighted by yellow boxes [11]. Peptidase M11 domain is shown by thick orange line and orange box in (A) and (B), respectively.

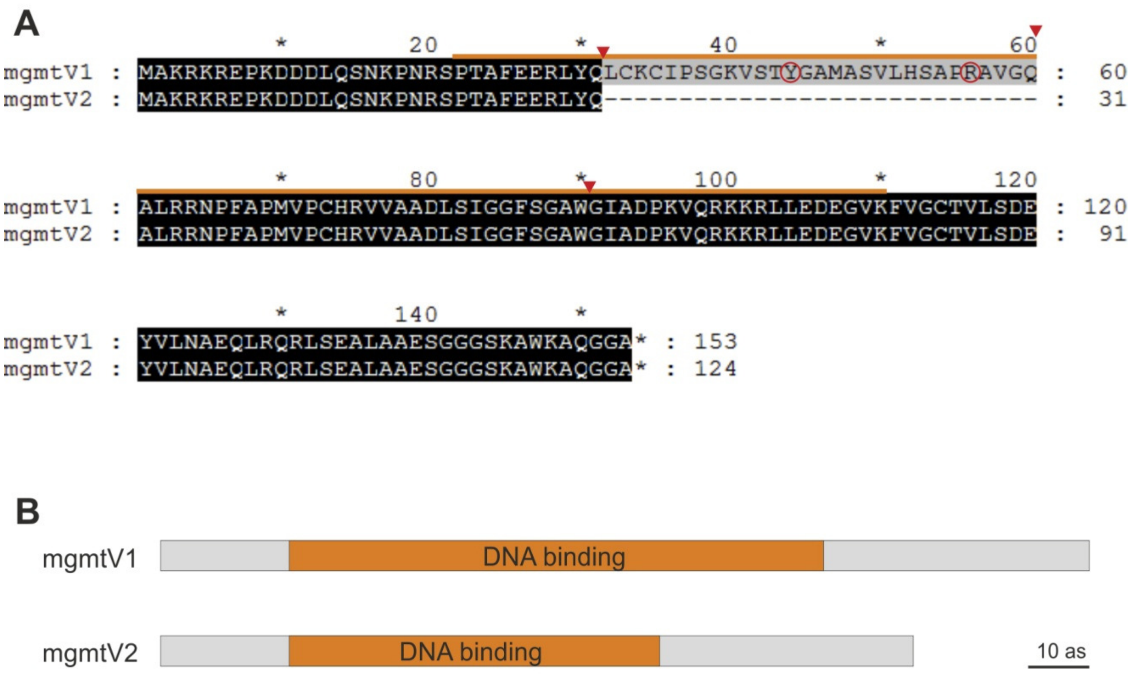

Figure S5: Comparison protein isoforms encoded by *mgmt*

Protein sequence alignment (A) and schematic representation (B) of the deduced amino acid sequences of two alternative splice variants (mgmtV1 and mgmtV2) of *mgmt*. DNA binding domain is shortened in mgmtV2. Two amino acid residues (Y44 and R57), which are important for DNA binding [12, 13], are highlighted by the red circles. Thick orange line and orange box represent the DNA binding domain in (A) and (B), respectively.

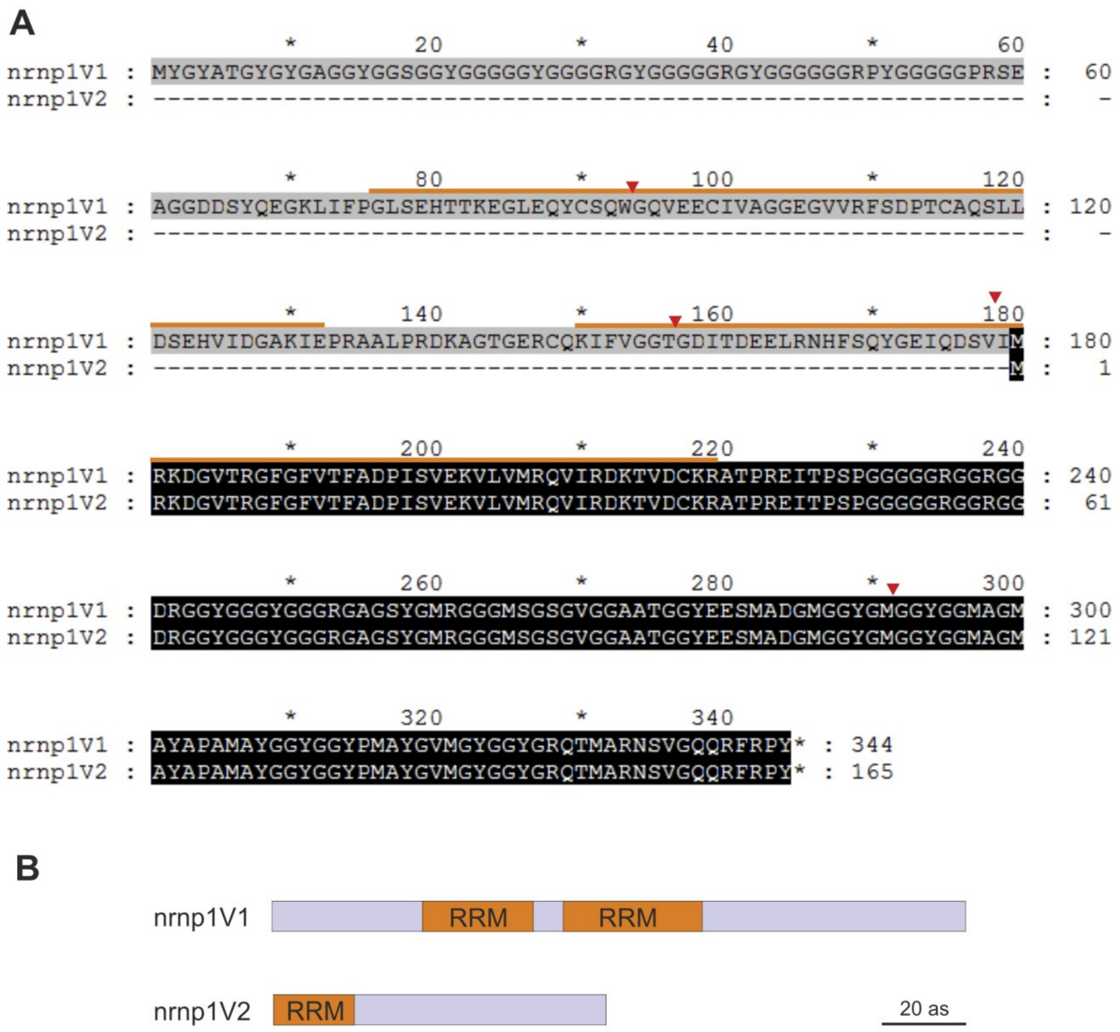

Figure S6: Comparison protein isoforms encoded by *nrnp1*

Protein sequence alignment (A) and schematic representation (B) of the deduced amino acid sequences of two alternative splice variants (nrnp1V1 and nrnp1V2) of *nrnp1*. nrnp1V1 contains two RNA binding domains. The first RNA binding domain and 30 amino acid residues from the second RRM domain are lacking in nrnp1V2. Both RRM domains are highlighted by orange thick line and orange box in (A) and (B), respectively.

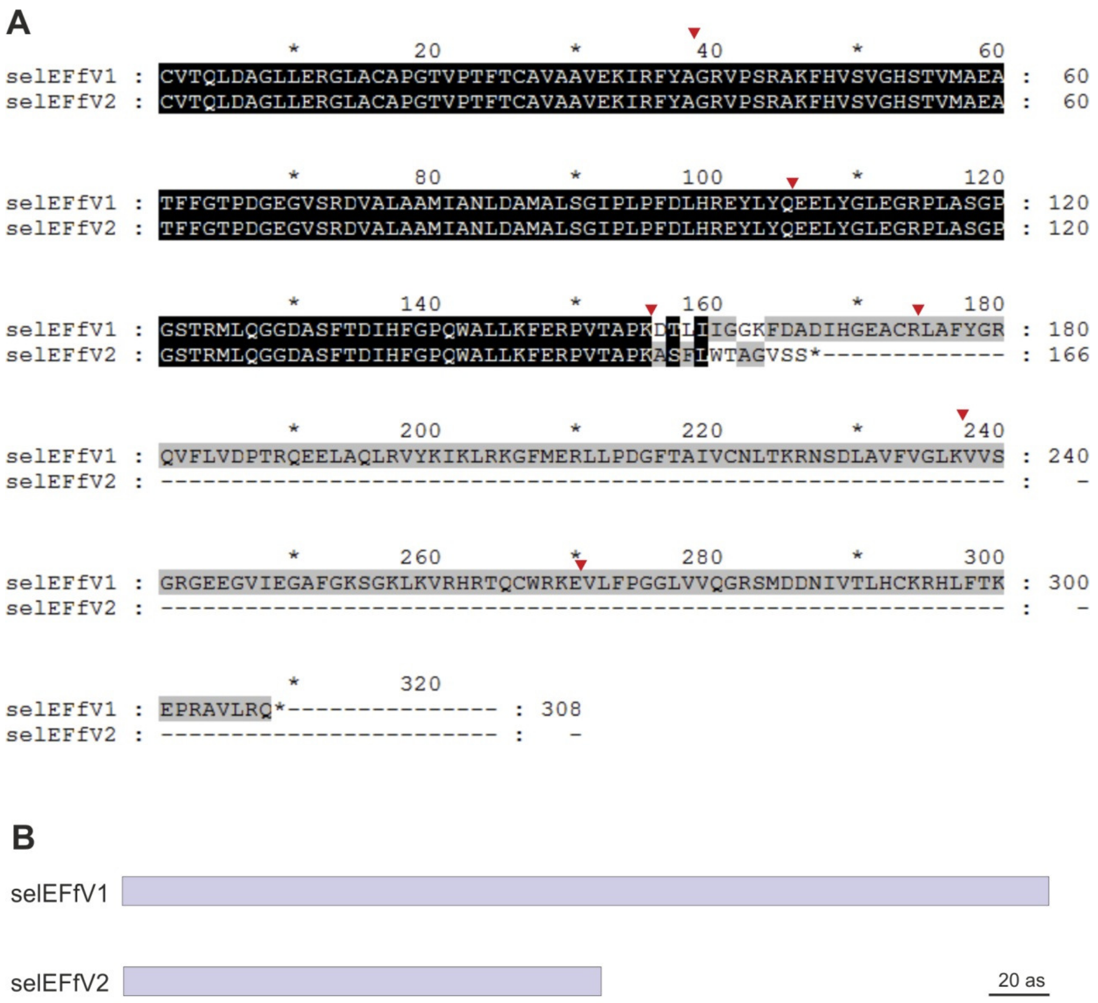

Figure S7: Comparison protein isoforms encoded by *selEFf*

Protein sequence alignment (A) and schematic representation (B) of the deduced amino acid sequences of two alternative splice variants (selEFfV1 and selEFfV2) of *selEFf*. The introduced premature stop codon by exon skipping leads to generate the second isoform (selEFfV2) which is 142 amino acids shorter than the first one (selEFfV1).

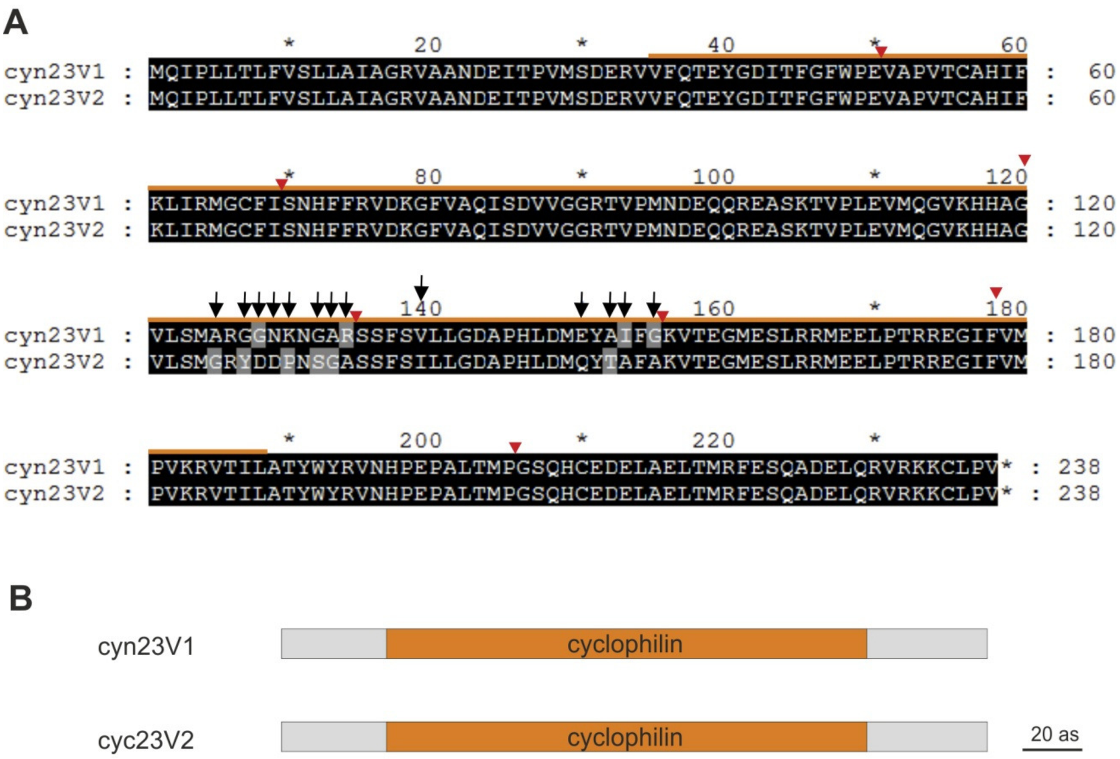

Figure S8: Comparison protein isoforms encoded by *cyn23*

Protein sequence alignment (A) and schematic representation (B) of the deduced amino acid sequences of two alternative splice variants (cyn23V1 and cyn23V2) of *cyn23*, which are produced by mutually exclusive exons alternative splicing (Figure 3 and Supplemental Figure S11). Both proteins are 238 amino acid residues in length and show differences in amino acid sequence. Arrows indicate the changes in amino acid sequences between cyn23V1 and cyn23V2. Thick orange line marked cyclophilin domain in (A), which is also highlighted by orange box in (B).

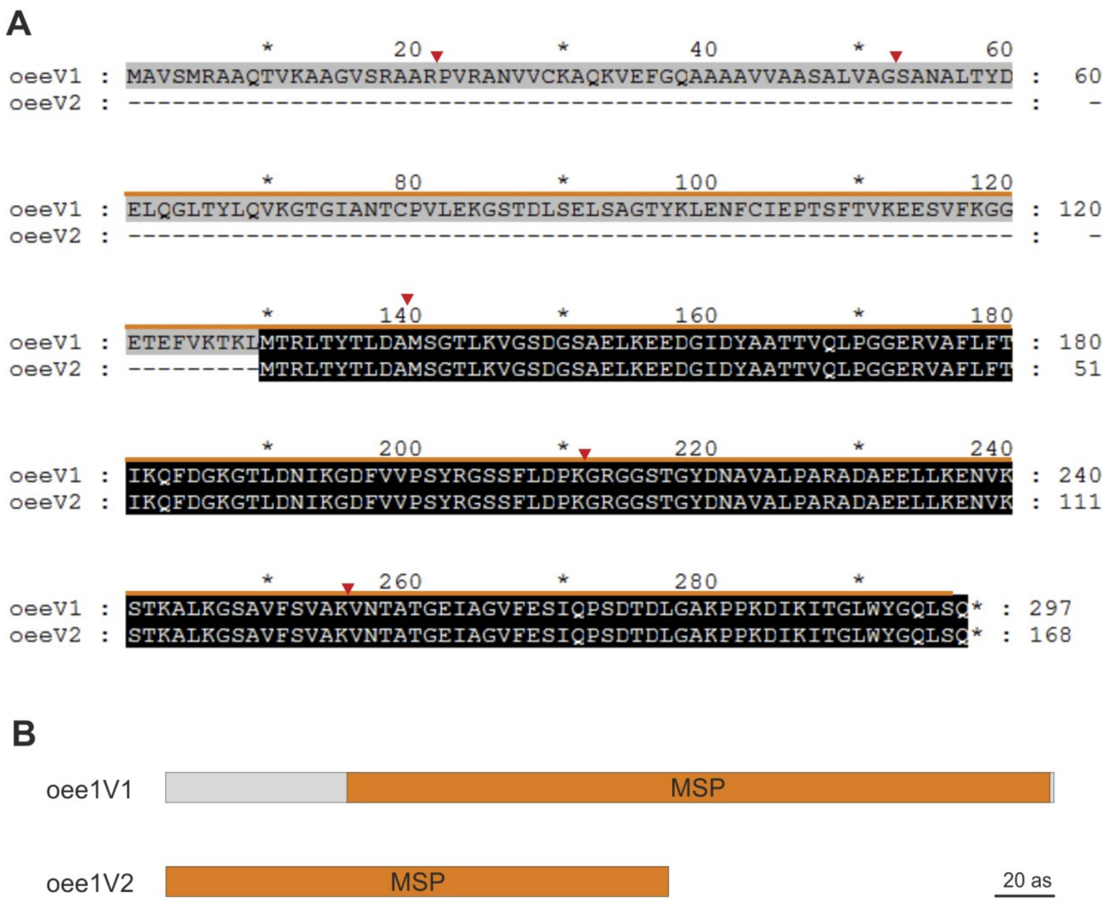

Figure S9: Comparison protein isoforms encoded by *oee1*

Protein sequence alignment (A) and schematic representation (B) of the deduced amino acid sequences of two alternative splice variants (oee1V1 and oee1V2) of *oee1*. A large part of manganese-stabilising protein (MSP) domain, which is highlighted by orange, is lacking in oee1V2. The domain is highlighted by thick orange line and orange box in (A) and (B), respectively.

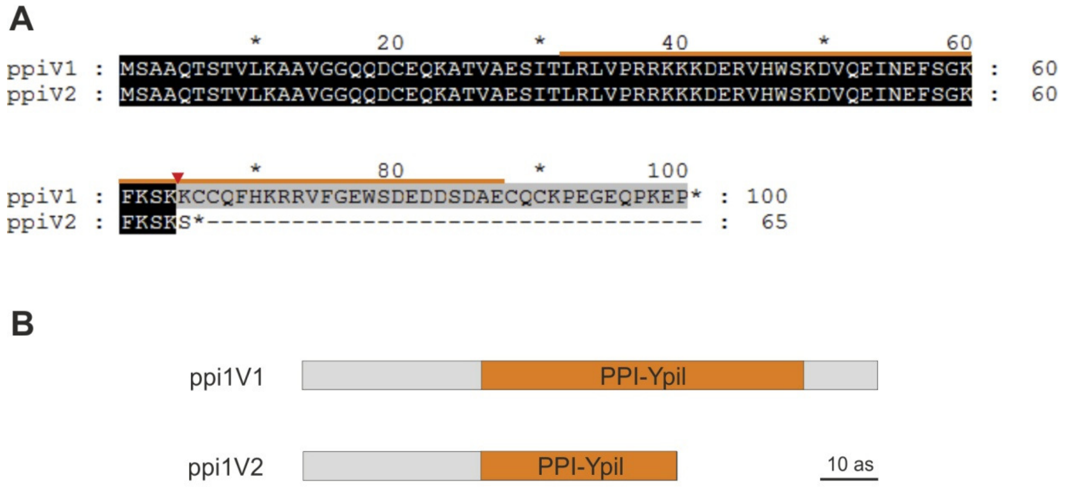

Figure S10: Comparison protein isoforms encoded by *ppi1*

Protein sequence alignment (A) and schematic representation (B) of the deduced amino acid sequences of two alternative splice variants (ppi1V1 and ppi1V2) of *ppi1*. The C-terminal truncated ppi1V2 lacks 23 amino acid residues of PPI-Ypil (Protein Phosphatase Type 1, heat-stable inhibitor of type 1 protein phosphatase) domain [14]. The PPI-Ypil domain is shown by thick orange line and orange box in (A) and (B), respectively.

```
cyn23V1      ATGCAGATCCCGTTATTAACGCTTTTTGTCTCATTATTGGCGATAGCTGGCAGAGTTGCT
cyn23V2      ATGCAGATCCCGTTATTAACGCTTTTTGTCTCATTATTGGCGATAGCTGGCAGAGTTGCT
             ************************************************************

cyn23V1      GCAAATGATGAGATCACCCCAGTTATGTCCGATGAACGCGTAGTGTTCCAAACGGAGTAT
cyn23V2      GCAAATGATGAGATCACCCCAGTTATGTCCGATGAACGCGTAGTGTTCCAAACGGAGTAT
             ************************************************************

cyn23V1      GGTGACATCACGTTCGGGTTTTGGCCAGAGGTGGCGCCGGTAACGTGCGCCCATATTTTC
cyn23V2      GGTGACATCACGTTCGGGTTTTGGCCAGAGGTGGCGCCGGTAACGTGCGCCCATATTTTC
             ************************************************************

cyn23V1      AAGCTAATCCGCATGGGTTGCTTTATCAGCAACCACTTTTTCCGCGTGGACAAGGGTTTC
cyn23V2      AAGCTAATCCGCATGGGTTGCTTTATCAGCAACCACTTTTTCCGCGTGGACAAGGGTTTC
             ************************************************************

cyn23V1      GTTGCGCAGATCTCTGATGTCGTAGGTGGTCGCACGGTTCCTATGAACGATGAGCAGCAG
cyn23V2      GTTGCGCAGATCTCTGATGTCGTAGGTGGTCGCACGGTTCCTATGAACGATGAGCAGCAG
             ************************************************************

cyn23V1      AGAGAGGCCTCCAAGACGGTGCCCCTCGAAGTCATGCAGGGCGTGAAGCACCATGCCGGC
cyn23V2      AGAGAGGCCTCCAAGACGGTGCCCCTCGAAGTCATGCAGGGCGTGAAGCACCATGCCGGC
             ************************************************************

cyn23V1      GTGCTTTCTATGGCGCGTGGAGGGAATAAGAACGGCGCACGGAGCAGCTTTTCGGTGCTG
cyn23V2      GTGTTATCGATGGGGCGCTATGATGACCCCAACTCGGGTGCCTCTAGCTTCAGCATTCTG
             *** * ** **** ***    *   *    ***   *      *****     * ***

cyn23V1      CTGGGCGACGCGCCGCATCTGGATATGGAGTATGCGATCTTTGGCAAGGTGACGGAGGGC
cyn23V2      CTGGGAGATGCACCGCACCTTGACATGCAGTACACCGCATTTGCCAAGGTGACGGAGGGC
             ***** ** ** ***** ** ** *** ****  *    **** ****************

cyn23V1      ATGGAGTCTCTGCGGCGGATGGAGGAGCTACCGACTAGGCGGGAGGGCATCTTCGTGATG
cyn23V2      ATGGAGTCTCTGCGGCGGATGGAGGAGCTACCGACTAGGCGGGAGGGCATCTTCGTGATG
             ************************************************************

cyn23V1      CCAGTGAAGCGTGTGACCATTCTTGCAACCTACTGGTACCGCGTCAACCACCCGGAGCCC
cyn23V2      CCAGTGAAGCGTGTGACCATTCTTGCAACCTACTGGTACCGCGTCAACCACCCGGAGCCC
             ************************************************************

cyn23V1      GCCTTGACGATGCCCGGTTCCCAGCATTGTGAGGATGAGCTTGCGGAGCTGACGATGCGG
cyn23V2      GCCTTGACGATGCCCGGTTCCCAGCATTGTGAGGATGAGCTTGCGGAGCTGACGATGCGG
             ************************************************************

cyn23V1      TTCGAAAGTCAGGCTGACGAGCTACAGCGGGTCCGCAAAAAGTGCCTGCCCGTTTGA
cyn23V2      TTCGAAAGTCAGGCTGACGAGCTACAGCGGGTCCGCAAAAAGTGCCTGCCCGTTTGA
             *********************************************************
```

Figure S11: cDNA alignment of *cyn23* splice variants

cDNA alignment of two splice variants of *cyn23*. Red and blue coloured nucleotides show exons 4 (106 bp in length) and exon 5 (106 bp in length) of *cyn23*-V1 and *cyn23*-V2, respectively (Figure 3H). Identical nucleotides are marked by asterisks.

A

Arabidopsis thaliana

EST number / in thousands	% AS events
176,915	1.5% [1]
278,734	11.6% [2]
369,218	21.8% [4]
756,761	30% [5]

***Volvox* (*V.c.*) and *Chlamydomonas* (*C.r.*)**

EST number / in thousands	% AS events
252,484 (*C.r.*)	3% [8]
132,038 (*V.c.*)	2.9% [this study]

B

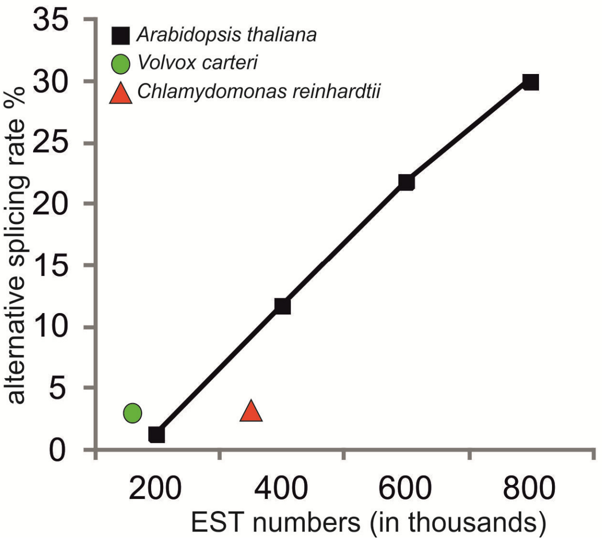

Figure S12: Relationship between estimated alternative splicing (AS) rate and EST coverage in *Arabidopsis thaliana*, *Volvox carteri* and *Chlamydomonas reinhardtii*

(**A**) The estimated AS rate in *Arabidopsis* correlates with the increase in the total number of sequenced ESTs. However, quite different EST numbers, i.e., 252,484 and 132,038, respectively, led to detect approximately the same alternative splice rate in both *Volvox* and *Chlamydomonas*. (**B**) Summary diagram of the relationship between alternative splicing rates and the total number of EST sequences.

Supplemental Tables

Table S1: Genome statistics of *Volvox* and other species

Comparison of the *Volvox carteri* genome with the genomes of *Chlamydomonas reinhardtii*, *Physcomitrella patens*, *Arabidopsis thaliana* and human.

organism	genome size	GC content	average gene size	average number of exons per gene	average number of introns per gene	average length of introns	average length of exons
Volvox carteri	138 Mb [15]	56% [15]	5.3 kb [15]	7.8 [15]	7.05 [15]	491 bp [15]	194 bp [15]
Chlamydomonas reinhardtii	118 Mb / 121 Mb [15, 16]	64% [16]	4.4 kb / 4.3 kb [15, 16]	8.4/8.3 [15, 16]	7.4 [15]	371 bp / 373 bp [15, 16]	232 bp / 190 bp [15, 16]
Physcomitrella patens	511 Mb [17]	38.7% [17]	2.4 kb [18]	4.9 [18]	NA*	311 bp [18]	246 bp [18]
Arabidopsis thaliana	125 Mb [19]	34.9% [19]	2 kb [19]	5.2 [19]	4.2 [19]	170 bp [19]	251 bp [19]
human	3,200 Mb [20]	41% [20]	27 kb [20]	8.8 [20]	7.8 [20]	3,365 bp [20]	145 bp [20]

*not available

Table S2: List of samples genes for experimental verification

List of ten selected alternatively spliced genes for investigation by RT-PCR and quantitative real-time RT-PCR. The first and second alternative splice variants are indicated as V1 and V2, respectively. ES, exon skipping; ME, mutually exclusive exons; IR, intron retention; Alt5´, alternative 5´ splice site.

gene	NCBI accession number	Gene length	Type of splicing	number of introns and exons in V1	number of introns and exons in V2	length of gene product in V1	length of gene product in V2	potentially modified domain by alternative splicing
clpr2	XP_002949433 [15]	2,599 bp	ES	8 exons 7 introns	7 exons 6 introns	284 aa	239 aa	caseinolytic protease (Clpr2P) PMID 9390554 [6]
efg8	ADI46910 [15, 21]	14,590 bp	ES	15 exons 14 introns	9 exons 8 introns	453 aa	283 aa	EF-Tu subfamily PMID 1542116, 7491491 and 11278992) [22-24]
hyd2	XP_002948483 [15]	4,388 bp	ES	9 exons 8 introns	7 exons 6 introns	497 aa	328 aa	hydrogenase, Fe-only PMID: 11070057 [25]
lsg2	BAB85218 [26]	8,333 bp	ES	12 exons 11 introns	11 exons 10 introns	625 aa	522 aa	gametolysin peptidase M11 PMID:12034745 and 11489172 [27, 28]
mgmt	XP_002948780 [15]	765 bp	ES	4 exons 3 introns	3 exons 2 introns	153 aa	124 aa	putative 6-O-methylguanine DNA methyltransferase PMID 15221026 [12]
nrnp1	XP_002954050 [15]	1,684 bp	ES	5 exons 4 introns	4 exons 3 introns	344 aa	165 aa	RRM (RNA recognition motif) PMID 10323862 [29]
selEFf	ADI46882 [21]	18,944 bp	ES	7 exons 6 introns	6 exons 5 introns	308 aa	166 aa	---
cyn23	XP_002953804 [15]	3,858 bp	ME	7 exons 6 introns	7 exons 6 introns	238 aa	238 aa	cyclophilin PMID 8652511 [30]
oee1	XP_002954867 [15]	1,553 bp	IR	6 exons 5 introns	5 exons 4 introns	297 aa	168 aa	manganese-stabilizing protein PMID: 3138527 and 16355230 [31, 32]
ppi1	XP_002956843 [15]	403 bp	Alt5´	2 exons 1 intron	2 exons 1 intron	100 aa	65 aa	protein phosphatase inhibitor PMID: 14506263 [14]

Table S3: Names as sequences of oligonucleotide primers

Names and sequences of oligonucleotide primers used to amplify alternative splicing variants of selected genes in RT-PCRs and quantitative real-time RT-PCRs (see Figure 4 for primer positions). The length of every splice variant is given in square brackets. The intron position is marked by an arrow if a primer spans an exon-exon junction.

gene name	specific primers for splice variant 1	specific primers for splice variant 2	specific primers to amplify both splice variants in the same RT-PCR reaction
clpr2	ON15265 (5'-AAGCGTAAGGTCACCACGATG) and ON15266 (5'-GCTTCTTTGATGAAGACCACCTTC) [134 bp]	ON15265 (5'-AAGCGTAAGGTCACCACGATG) and ON15267 (5'-GAGGGTTGGAACAAC↓GTAGG) [117 bp]	ON15265 (5'-AAGCGTAAGGTCACCACGATG) and ON15371 (5'-ACGTATTGCATCGTGTCGTACAG) [278 bp (V1) and 143 bp (V2)]
efg8	ON15273 (5'-CACCAATCGCCACTATGCTC) and ON15274 (5'-CAAGAGAATGTGCTCACGAGTC) [151 bp]	ON15271 (5'-CAATTAATTCCACTCACG↓GAGTC) and ON15272 (5'-CACGTCCTCACGCTTTATACC) [176 bp]	ON15365 (5'-GTCCACAAAGGCTGTTTCTTACG) and ON15366 (5'-ACCAGTGACTGTGCTCTTAATCG) [664 bp (V1) and 154 bp (V2)]
hyd2	ON15275 (5'-GGTGAAGACGGTGCAAGAAG) and ON15276 (5'-TGCGCTCAGCCTTTGGCTTG) [128 bp]	ON15275 (5'-GGTGAAGACGGTGCAAGAAG) and ON15277 (5'-GAGCCTGAAGCCTGCT↓TCCA) [102 bp]	ON15275 (5'-GGTGAAGACGGTGCAAGAAG) and ON15362 (5'-GGCAGCAGCTAGTAAACATGG) [386 bp (V1) and 159 bp (V2)]
lsg2	ON15294 (5'-GGTCATCTTGCATCGTGTACATC) and ON15295 (5'-CATGACGTCGGAGAAATCGC) [137 bp]	ON15294 (5'-GGTCATCTTGCATCGTGTACATC) and ON15296 (5'-GAAGTTGTGCACGAAGAC↓CTCA) [131 bp]	ON15294 (5'-GGTCATCTTGCATCGTGTACATC) and ON15368 (5'-CAGCAGCGTACGGTTGTAATC) [482 bp (V1) and 173 bp (V2)]
mgmt	ON15278 (5'-GTGTAAAGGCTGATCTCGAGAAAG) and ON15279 (5'-CAGACGGGATACACTTGCAAAG) [173 bp]	ON15278 (5'-GTGTAAAGGCTGATCTCGAGAAAG) and ON15280 (5'-TTCCGGCGAAGTGC↓CTGGT) [165 bp]	ON15278 (5'-GTGTAAAGGCTGATCTCGAGAAAG) and ON15367 (5'-TCCTATGCTGAGGTCTGCAG) [310 bp (V1) and 223 bp (V2)]
nrnp1	ON15286 (5'-TGACGACTCGTACCAGGAGG) and ON15287 (5'-CTACGATGCACTCCTCGACC) [116 bp]	ON15286 (5'-TGACGACTCGTACCAGGAGG) and ON15288 (5'-TCGTCCGTAATATCTCC↓CACTG) [107 bp]	ON15286 (5'-TGACGACTCGTACCAGGAGG) and ON15370 (5'-GAGTCTTGAATCTCTCCATACTGAG) [342 bp (V1) and 152 bp (V2)]
selEFf	ON15289 (5'-AGGAGCTGTACGGCTTGGAG) and ON15290 (5'-TCACCGTGTATATCCGCGTC) [196 bp]	ON15289 (5'-AGGAGCTGTACGGCTTGGAG) and ON15291 (5'-GTCCATAGAAAGCTAGC↓CTTGG) [166 bp]	ON15289 (5'-AGGAGCTGTACGGCTTGGAG) and ON15369 (5'-TCGTGAGGTTGCACACAATAGC) [357 bp (V1) and 301 bp (V2)]
cyn23	ON15268 (5'-CACGGTTCCTATGAACGATGAG) and ON15269 (5'-CACCGAAAAGCTGCTCCGTG) [145 bp]	ON15268 (5'-CACGGTTCCTATGAACGATGAG) and ON15270 (5'-GAATGCTGAAGCTAGAGGCAC) [146 bp]	---
oee1	ON15281 (5'-CTTCGCTTCCTTTGCCAATTACC) and ON15285 (5'-TTGGCACGGACCGGC↓CTGGCAG) [111 bp]	ON15281 (5'-CTTCGCTTCCTTTGCCAATTACC) and ON15284 (5'-GGAGTTACGATATGGCTATGGTC) [158 bp]	ON15281 (5'-CTTCGCTTCCTTTGCCAATTACC) and ON15364 (5'-GATGCAGAAGTTCTCCAGCTTG) [346 bp (V1) and 415 bp (V2)]
ppi1	ON15300 (5'-CAGTAGCAGAGTCCATTACATTGC) and ON15302 (5'-CTTATGAAACTGACAGCACT↓TCTTC) [140 bp]	ON15300 (5'-CAGTAGCAGAGTCCATTACATTGC) and ON15301 (5'-CTGAAAGCCTCAGCACCTAAC) [141 bp]	ON15300 (5'-CAGTAGCAGAGTCCATTACATTGC) and ON15363 (5'-ATGGCTCCTTAGGTTGCTCTC) [228 bp (V1) and 249 bp (V2)]

Table S4: Alternative splicing status of orthologous genes between *Volvox* and its close relative *Chlamydomonas*

Names and sequences of oligonucleotide primers used to amplify alternative splicing variants of selected genes in RT-PCRs and quantitative real-time RT-PCRs (see Figure 4 for primer positions). The length of every splice variant is given in square brackets. The intron position is marked by an arrow if a primer spans an exon-exon junction.

Blue background: 10 alternatively spliced genes selected for experimental verification in this study
Yes (green background): genes with alternative splicing events
No (red background): no alternative splicing events were identified
Numbers: represent the EST coverage for those genes that do not exhibit alternative splice events in *Chlamydomonas* based on available data
(http://phytozome.jgi.doe.gov/pz/portal.html#!info?alias=Org_Creinhardtii)

Gene model	Gene description / accession number in *Volvox*	Gene description / accession number in *Chlamydomonas*	alternatively spliced in *Volvox*	alternatively spliced in *Chlamydomonas*
1	chloroplast Clp protease (clpr2) / XM_002949387	non-catalytic subunit of chloroplast ClpP complex / EDP04400	Yes	No (~50)
2	mitochondrial translation elongation factor Tu (efg8) / XM_002958233	mitochondrial translation factor Tu, putative mitochondrial translation factor Tu / EDP08321	Yes	Yes
3	iron hydrogenase (hyd2) / XM_002948437	iron hydrogenase / EDP02498	Yes	Yes
4	matrix metalloproteinase (lsg2) / BAB85218	Gametolysin peptidase M11 / EDP02375	Yes	No (~100)
5	6-O-methylguanine DNA methyltransferase (mgmt) / EFJ50160	Methylated-DNA-[protein]-cysteine S-methyltransferase / EDP01091	Yes	No (~30)
6	nuclear ribonucleoprotein (nrnp1) / EFJ44767	nuclear ribonucleoprotein / EDP06492	Yes	No (~50)
7	selenocysteine-specific elongation factor (selEFf) / ADI46882	selenocysteine-specific elongation factor EF-Sec / EDP08320	Yes	Yes
8	cyclophilin-type peptidyl-prolyl cis-trans isomerase (cyn23) / EFJ45128	cyclophilin-like protein / XP_001701608	Yes	No (~40)
9	oxygen evolving enhancer protein 1 (oee1) / AF110780	Oxygen-evolving enhancer protein 1 / P12853	Yes	No (~80)
10	protein phosphatase inhibitor (ppi1) / XM_002956797	flagellar associated protein, protein phosphatase inhibitor / EDO97732	Yes	Yes
11	eukaryotic translation elongation factor 1 alpha 2 / XM_002945745	eukaryotic translation elongation factor 1 alpha 2 / EDO96967	Yes	No (~50)
12	leucine rich repeats / XP_002945949	flagellar associated protein, leucine rich repeat / EDP08219	Yes	No (26)
13	RNA-binding protein / XP_002945692	nucleic acid binding protein / EDP09553	Yes	Yes
14	arogenate/prephenate dehydrogenase / XP_002945921	arogenate/prephenate dehydrogenase / EDP01548	Yes	No (~50)
15	leucine-rich repeat receptor-like protein kinase / XP_002945643	leucine-rich repeat receptor-like protein kinase / EDO99036	Yes	Yes
16	20S proteasome alpha subunit E / EFJ52696	20S proteasome alpha subunit E / EDP00486	Yes	No (~60)
17	leucine rich repeat / EFJ52641	coiled-coil domain-containing protein lobo homolog / A8JAM0	Yes	No (~100)
18	hypothetical protein VOLCADRAFT_102812 / EDP08472	predicted protein / EDP08472	Yes	Yes
19	ribosomal protein S18 / EFJ53218	埝 吋栀somal protein S18 / EDP08449	Yes	No (~30)
20	plastid/chloroplast ribosomal protein L28 / EFJ53271	plastid ribosomal protein L28 / EDP08523	Yes	No (~50)
21	ribosomal protein L21 / EFJ53106	ribosomal protein L21 / EDO99609	Yes	No (~40)
22	hypothetical protein VOLCADRAFT_102758 / EFJ53137	predicted protein / EDP08274	Yes	No (~30)

23	nucleic acid binding protein nab1 / ABM47305	nucleic acid binding protein nab1 / EDP08495	Yes	No (~70)
24	bardet-Biedl syndrome 5 / EFJ53283	bardet-biedl syndrome 5 protein / EDP08230	Yes	Yes
25	Oligosaccharyltransferase, beta subunit / EFJ53013	dolichyl-diphosphooligosaccharide-protein glycosyltransferase / EDO99098	Yes	No (~50)
26	nucleolar protein involved in 40S ribosome biogenesis / EFJ53161	nucleolar protein / EDP08566	Yes	Yes
27	component of cytosolic 80S ribosome and 60S large subunit / EFJ52307	ribosomal protein L15 / EDP06755	Yes	No (~40)
28	hypothetical protein VOLCADRAFT_72703, RNA-binding protein / EFJ52313	predicted protein, RNA-binding protein / EDP06734	Yes	No (~30)
29	component of cytosolic 80S ribosome and 60S large subunit / EFJ52575	ribosomal protein L35 / EDP09570	Yes	No (~50)
30	putative signal transduction protein p25 / EFJ52595	flagellar associated protein / EDP02168	Yes	Yes
31	AP_delta-COPI_MHD / EFJ52476	delta-cop / EDP06738	Yes	No (~60)
32	hypothetical protein VOLCADRAFT_102959 / EFJ52464	hypothetical protein CHLREDRAFT_141551 / EDP06961	Yes	No (~30)
33	serine/threonine phosphatases / EFJ40674	serine/threonine protein phosphatase / EDP08334	Yes	Yes
34	component of cytosolic 80S ribosome and 40S small subunit / EFJ51026	ribosomal protein S7 / EDO96962	Yes	No (~60)
35	tubulin alpha-1/alpha-2 chain / P11481	tubulin alpha-1 chain / P09204	Yes	No (~30)
36	nucleosome assembly protein (NAP) / EFJ42879	nucleosome assembly protein / EDP00863	Yes	No (~50)
37	hypothetical protein VOLCADRAFT_82819, putative substrate binding site / EFJ44426	predicted protein, putative ATP binding site / EDP08635	Yes	No (~30)
38	tubulin beta chain / P11482	Tubulin beta-1/beta-2 chain / P04690	Yes	No (~50)
39	Peptide methionine sulfoxide reductase / EFJ40180	peptide methionine sulfoxide reductase / EDP09617	Yes	No (~60)
40	light-harvesting protein of photosystem I / EFJ40348	light-harvesting protein of photosystem I / EDP01593	Yes	No (~60)
41	hypothetical protein VOLCADRAFT_107753 / EFJ41282	predicted protein / EDO99878	Yes	No (~60)
42	hypothetical protein VOLCADRAFT_107125 / EFJ42773	predicted protein / EDP09346	Yes	Yes
43	component of cytosolic 80S ribosome and 40S small subunit / EFJ43680	ribosomal protein S10 / EDP00807	Yes	Yes
44	Pherophorin / EFJ45544	cell wall protein pherophorin-C1 / EDO95895	Yes	No (~60)
45	eukaryotic translation initiation factor 5A / EFJ48283	eukaryotic initiation factor / EDP07536	Yes	No (~50)
46	hypothetical protein VOLCADRAFT_74648, PsbP domain-containing protein / EFJ48537	predicted protein, PsbP domain-containing protein 4 / EDP03267	Yes	No (~60)
47	heat shock protein 70B / EFJ40669	heat shock protein 70B / EDP08409	Yes	No (~50)
48	s-adenosylmethionine synthase / ADI46846	s-adenosylmethionine synthase / A8HYU5	Yes	No (~70)
49	ribulose-bisphosphate carboxylase small subunit / XP_002955684	ribulose bisphosphate carboxylase small chain 1 / P00873	Yes	Yes
50	hypothetical protein VOLCADRAFT_107619, CCC1-related family of proteins / EFJ41666	predicted protein, nodulin-21 and CCC1-related protein family / EDP07718	Yes	Yes
51	F1F0 ATP synthase, subunit C / EFJ42381	F1F0 ATP synthase subunit 9 / EDO97408	Yes	Yes
52	hypothetical protein VOLCADRAFT_75409, phosphopantetheine attachment site / XP_002952514	acyl-carrier protein / EDP09036	Yes	No (~30)
53	plastid/chloroplast ribosomal protein L35 / EFJ47858	plastid ribosomal protein L35 / EDO97643	Yes	No (16)
54	hypothetical protein VOLCADRAFT_106042 , Pleckstrin	predicted protein, alpha-ketoglutarate decarboxylase / EDP06665	Yes	No (~30)

	homology-like domain / EFJ45228			
55	hypothetical protein VOLCADRAFT_105932, OST3 / OST6 family / EFJ45380	predicted membrane protein, OST3-OST6 family / EDP00559	Yes	No (~30)
56	hypothetical protein VOLCADRAFT_107666, HAT (Half-A-TPR) repeats / XP_002957409	nuclear pre-mRNA splicing factor and U1 snRNP component / EDO98818	Yes	Yes
57	axonemal dynein light chain / EFJ42000	flagellar inner arm dynein light chain p28 / EDO97971	Yes	No (~30)
58	DEAD-box RNA helicase, ATP-dependent / EFJ43482	DEAD-box RNA helicase / EDP09760	Yes	No (~50)
59	hypothetical protein VOLCADRAFT_105833, ParB-like nuclease domain /EFJ45749	sulfiredoxin / EDO97150	Yes	No (13)
60	hypothetical protein VOLCADRAFT_104697, HR-like lesion-inducin / EFJ48515	predicted protein, HR-like lesion-inducing / EDP04339	Yes	No (~50)
61	hypothetical protein VOLCADRAFT_104422, FUN14 family / EFJ49205	predicted protein, FUN14 family / EDP04838	Yes	Yes
62	long-chain acyl-CoA synthetases / XP_002949145	long-chain-fatty-acid CoA ligase / EDP05022	Yes	No (~60)
63	hypothetical protein VOLCADRAFT_84253, WD40 repeat / EFJ40989	U5 snRNP-specific protein / EDP08353	Yes	No (~50)
64	hypothetical protein VOLCADRAFT_98257, large tegument protein UL36 / EFJ41679	predicted protein / EDP03306	Yes	No (~70)
65	hypothetical protein VOLCADRAFT_76090,Thioredoxin (TRX) domain containing protein 9 / XP_002953944	thioredoxin-like ATP binding protein 2 / EDP09856	Yes	No (~30)
66	hypothetical formate nitrite transporter / EFJ48615	formate nitrite transporter / EDO98442	Yes	Yes
67	Ubiquinol-cytochrome C reductase hinge protein / EFJ49241	ubiquinol:cytochrome c oxidoreductase 8 kDa subunit / EDO99747	Yes	Yes
68	intraflagellar transport protein IFT20 / EFJ49364	intraflagellar transport particle protein 20 / EDP06941	Yes	Yes
69	hypothetical protein VOLCADRAFT_120666, RNA recognition motif / EFJ50216	SR protein factor / EDP08875	Yes	No (~40)
70	uroporphyrinogen decarboxylase chloroplast precursor / EFJ50429	uroporphyrinogen decarboxylase / EDP06796	Yes	No (~40)
71	hypothetical protein VOLCADRAFT_120394, cAMP-regulated phosphoprotein/endosulfine conserved / EFJ52215 region	predicted protein, Chromosome segregation ATPases / EDP04959	Yes	No (~100)
72	radial spokehead-like protein / EFJ40102	flagellar radial spoke protein 4 / Q01656	Yes	Yes
73	hypothetical protein, type III pyridoxal 5-phosphate (PLP)-dependent / XM_002958599	ornithine decarboxylase / AJ581668	Yes	Yes
74	triose-phosphate Transporter family / XM_002958091	triose phosphate transporter / AY177787	Yes	No (~60)
75	component of cytosolic 80S ribosome and 40S small subunit / EFJ44096	ribosomal protein S4 / EDP04951	Yes	No (~30)
76	hypothetical protein, chlorophyll A-B binding protein / EFJ44207	early light-inducible protein / EDP02335	Yes	No (~30)
77	hypothetical protein / EFJ44711	predicted protein / EDP07079	Yes	No (17)
78	cyclophilin_ABH_like / EFJ45179	peptidyl-prolyl cis-trans isomerase, cyclophilin-type / EDO98214	Yes	No (~30)
79	hypothetical protein, ribosome associated membrane protein / EFJ44791	predicted protein, ribosome associated membrane protein / EDP00157	Yes	No (~30)
80	hypothetical protein, cytochrome b6f complex subunit 7 / EFJ45349	cytochrome b6-f complex subunit 7 / Q42496	Yes	No (~30)
81	hypothetical protein, K homology RNA-	predicted protein, nucleic acid binding	Yes	No (~30)

	binding domain / EFJ46104	region / EDP01252		
82	hypothetical protein / EFJ47103	predicted protein, chloroplast location proposed / XP_001703377	Yes	Yes
83	hypothetical protein VOLCADRAFT_105076 /EFJ47604	predicted protein / EDO99963	Yes	Yes
84	ferredoxin like, hypothetical	ferredoxin, adrenodoxin-like protein / EDO96620	Yes	No (~30)
85	hypothetical protein, protein Kinases, catalytic domain / XP_002948316	glycogen synthase kinase 3 / EDP05327	Yes	Yes
86	hypothetical protein / EFJ51470	predicted protein / EDP03488	Yes	Yes
87	ATP synthase subunit D / EFJ51827	vacuolar ATP synthase subunit D / EDP01852	Yes	No (~30)
88	hypothetical protein, archease protein family / EFJ53137	predicted protein, archease protein family / EDP08274	Yes	No (~30)
89	hypothetical protein, pyridoxamine 5'-phosphate oxidase / EFJ40229	predicted protein, pyridoxamine 5'-phosphate oxidase / EDP07732	Yes	No (~50)
90	NADH:ubiquinone oxidoreductase 18 kDa subunit / EFJ40445	NADH:ubiquinone oxidoreductase 18 kDa subunit / EDP01388	Yes	Yes
91	ALB3_1f / ADI46852	inner membrane ALBINO3-like protein 1 / EDP08623	Yes	No (~40)
92	hypothetical protein, pyruvate formate lyase 1 / EFJ40790	pyruvate-formate lyase / EDP09457	Yes	No (~70)
93	component of cytosolic 80S ribosome and 40S small subunit /EFJ41382	ribosomal protein S12 / EDO99351	Yes	Yes
94	hypothetical protein, Kelch domain /EFJ41755	predicted protein, Kelch domain / EDP03274	Yes	No (~50)
95	hypothetical protein / EFJ41890	predicted protein / EDP07800	Yes	No (~30)
96	hypothetical protein, WD40 domain / EFJ43030	guanine nucleotide-binding protein / EDP05920	Yes	No (~30)
97	bola-like protein / EFJ43764	BolA-like protein / EDP02592	Yes	No (~30)
98	hypothetical protein, tocopherol O-methyltransferase / EFJ46509	N-myristoyl transferase / EDP08632	Yes	No (~40)
99	hypothetical protein, branched-chain-amino-acid aminotransferase-like protein / EFJ50280	aminotransferase / EDP08786	Yes	Yes
100	putative FKBP-type peptidyl-prolyl cis-trans isomerases 1 / EFJ50220	peptidyl-prolyl cis-trans isomerase, FKBP-type / EDP08869	Yes	No (~40)

Supplemental References

1. Zhu W, Schlueter SD, Brendel V: **Refined annotation of the *Arabidopsis* genome by complete expressed sequence tag mapping**. *Plant Physiol* 2003, **132**(2):469-484.
2. Iida K, Seki M, Sakurai T, Satou M, Akiyama K, Toyoda T, Konagaya A, Shinozaki K: **Genome-wide analysis of alternative pre-mRNA splicing in *Arabidopsis thaliana* based on full-length cDNA sequences**. *Nucleic Acids Res* 2004, **32**(17):5096-5103.
3. Nicholas KB, Nicholas HB, Deerfield DW: **GeneDoc: Analysis and visualization of genetic variation**. *EMBnetnews* 1997, **4**:14.
4. Wang BB, Brendel V: **Genomewide comparative analysis of alternative splicing in plants**. *Proc Natl Acad Sci U S A* 2006, **103**(18):7175-7180.
5. Campbell MA, Haas BJ, Hamilton JP, Mount SM, Buell CR: **Comprehensive analysis of alternative splicing in rice and comparative analyses with *Arabidopsis***. *BMC Genomics* 2006, **7**:327.
6. Wang J, Hartling JA, Flanagan JM: **The structure of ClpP at 2.3 Å resolution suggests a model for ATP-dependent proteolysis**. *Cell* 1997, **91**(4):447-456.
7. Bourne HR, Sanders DA, McCormick F: **The GTPase superfamily: conserved structure and molecular mechanism**. *Nature* 1991, **349**(6305):117-127.
8. Labadorf A, Link A, Rogers MF, Thomas J, Reddy AS, Ben-Hur A: **Genome-wide analysis of alternative splicing in *Chlamydomonas reinhardtii***. *BMC Genomics* 2010, **11**:114.
9. Peters JW, Lanzilotta WN, Lemon BJ, Seefeldt LC: **X-ray crystal structure of the Fe-only hydrogenase (CpI) from *Clostridium pasteurianum* to 1.8 Angstrom resolution**. *Science* 1998, **282**(5395):1853-1858.
10. Forestier M, King P, Zhang L, Posewitz M, Schwarzer S, Happe T, Ghirardi ML, Seibert M: **Expression of two [Fe]-hydrogenases in *Chlamydomonas reinhardtii* under anaerobic conditions**. *Eur J Biochem* 2003, **270**(13):2750-2758.
11. Hooper NM: **Families of zinc metalloproteases**. *FEBS Lett* 1994, **354**(1):1-6.
12. Daniels DS, Woo TT, Luu KX, Noll DM, Clarke ND, Pegg AE, Tainer JA: **DNA binding and nucleotide flipping by the human DNA repair protein AGT**. *Nat Struct Mol Biol* 2004, **11**(8):714-720.
13. Duguid EM, Rice PA, He C: **The structure of the human AGT protein bound to DNA and its implications for damage detection**. *J Mol Biol* 2005, **350**(4):657-666.
14. García-Gimeno MA, Muñoz I, Ariño J, Sanz P: **Molecular characterization of Ypi1, a novel *Saccharomyces cerevisiae* type 1 protein phosphatase inhibitor**. *J Biol Chem* 2003, **278**(48):47744-47752.
15. Prochnik SE, Umen J, Nedelcu AM, Hallmann A, Miller SM, Nishii I, Ferris P, Kuo A, Mitros T, Fritz-Laylin LK *et al*: **Genomic analysis of organismal complexity in the multicellular green alga *Volvox carteri***. *Science* 2010, **329**(5988):223-226.
16. Merchant SS, Prochnik SE, Vallon O, Harris EH, Karpowicz SJ, Witman GB, Terry A, Salamov A, Fritz-Laylin LK, Marechal-Drouard L *et al*: **The *Chlamydomonas* genome reveals the evolution of key animal and plant functions**. *Science* 2007, **318**(5848):245-250.
17. Lang D, Zimmer AD, Rensing SA, Reski R: **Exploring plant biodiversity: the *Physcomitrella* genome and beyond**. *Trends Plant Sci* 2008, **13**(10):542-549.
18. Rensing SA, Lang D, Zimmer AD, Terry A, Salamov A, Shapiro H, Nishiyama T, Perroud PF, Lindquist EA, Kamisugi Y *et al*: **The *Physcomitrella* genome reveals evolutionary insights into the conquest of land by plants**. *Science* 2008, **319**(5859):64-69.
19. Arabidopsis Genome Initiative: **Analysis of the genome sequence of the flowering plant *Arabidopsis thaliana***. *Nature* 2000, **408**(6814):796-815.
20. Lander ES, Linton LM, Birren B, Nusbaum C, Zody MC, Baldwin J, Devon K, Dewar K, Doyle M, FitzHugh W *et al*: **Initial sequencing and analysis of the human genome**. *Nature* 2001, **409**(6822):860-921.

21. Ferris P, Olson BJ, De Hoff PL, Douglass S, Casero D, Prochnik S, Geng S, Rai R, Grimwood J, Schmutz J *et al*: **Evolution of an expanded sex-determining locus in *Volvox***. *Science* 2010, **328**(5976):351-354.
22. Kjeldgaard M, Nyborg J: **Refined structure of elongation factor EF-Tu from *Escherichia coli***. *J Mol Biol* 1992, **223**(3):721-742.
23. Nissen P, Kjeldgaard M, Thirup S, Polekhina G, Reshetnikova L, Clark BF, Nyborg J: **Crystal structure of the ternary complex of Phe-tRNAPhe, EF-Tu, and a GTP analog**. *Science* 1995, **270**(5241):1464-1472.
24. Vogeley L, Palm GJ, Mesters JR, Hilgenfeld R: **Conformational change of elongation factor Tu (EF-Tu) induced by antibiotic binding. Crystal structure of the complex between EF-Tu.GDP and aurodox**. *J Biol Chem* 2001, **276**(20):17149-17155.
25. Horner DS, Foster PG, Embley TM: **Iron hydrogenases and the evolution of anaerobic eukaryotes**. *Mol Biol Evol* 2000, **17**(11):1695-1709.
26. Shimizu T, Inoue T, Shiraishi H: **Cloning and characterization of novel extensin-like cDNAs that are expressed during late somatic cell phase in the green alga *Volvox carteri***. *Gene* 2002, **284**(1-2):179-187.
27. Heitzer M, Hallmann A: **An extracellular matrix-localized metalloproteinase with an exceptional QEXXH metal binding site prefers copper for catalytic activity**. *J Biol Chem* 2002, **277**(31):28280-28286.
28. Hallmann A, Amon P, Godl K, Heitzer M, Sumper M: **Transcriptional activation by the sexual pheromone and wounding: a new gene family from *Volvox* encoding modular proteins with (hydroxy)proline-rich and metalloproteinase homology domains**. *Plant J* 2001, **26**(6):583-593.
29. Ding J, Hayashi MK, Zhang Y, Manche L, Krainer AR, Xu RM: **Crystal structure of the two-RRM domain of hnRNP A1 (UP1) complexed with single-stranded telomeric DNA**. *Genes Dev* 1999, **13**(9):1102-1115.
30. Zhao Y, Ke H: **Crystal structure implies that cyclophilin predominantly catalyzes the trans to cis isomerization**. *Biochemistry* 1996, **35**(23):7356-7361.
31. Philbrick JB, Zilinskas BA: **Cloning, nucleotide sequence and mutational analysis of the gene encoding the Photosystem II manganese-stabilizing polypeptide of *Synechocystis* 6803**. *Mol Gen Genet* 1988, **212**(3):418-425.
32. Loll B, Kern J, Saenger W, Zouni A, Biesiadka J: **Towards complete cofactor arrangement in the 3.0 Å resolution structure of photosystem II**. *Nature* 2005, **438**(7070):1040-1044.
